# Cellular imbalance in proximal and distal lung of *CFTR*^*−/−*^ sheep in utero and at birth

**DOI:** 10.1186/s10020-025-01266-7

**Published:** 2025-06-11

**Authors:** Shih-Hsing Leir, Svyatoslav Tkachenko, Alekh Paranjapye, Arnaud J. Van Wettere, Jenny L. Kerschner, Iuri Viotti Perisse, Cheyenne M. Marriott, Tayler Patrick, Ying Liu, Kenneth L. White, Irina A. Polejaeva, Ann Harris

**Affiliations:** 1 Department of Genetics and Genome Sciences, Case Western Reserve University School of Medicine, Cleveland, OH 44106-4955 USA; 2 Present Address: Department of Genetics, University of Pennsylvania, Philadelphia, PA USA; 3 Department of Animal, Dairy and Veterinary Sciences, Utah State University, Logan, UT USA

**Keywords:** Cystic fibrosis (CF), In utero, Lung development, ScRNA-seq, Ciliated cells

## Abstract

**Background:**

The Lung is the major focus of therapeutic approaches for the inherited disorder cystic fibrosis (CF) as without treatment lung disease is life-limiting. However, the initiating events that predispose the CF lung to cycles of infection, inflammation and resultant tissue damage are still unclear. Inflammation may occur in the CF lung prior to birth in human and several large animal models suggesting an in utero origin for the disease and encouraging further studies prior to birth.

**Methods:**

Here we used the sheep model of CF (*CFTR*^*−/−*^) and age-matched wild-type (WT) sheep of the same breed to investigate the single cell transcriptomes of proximal and distal lung tissue at 80 days and 120 days of gestation and at term (147 days). Single cell RNA-seq was performed on tissues from 4 to 7 animals of each genotype (WT and *CFTR*^*−/−*^) at each time point.

**Results:**

At term, FOXJ1-expressing ciliated cells are overrepresented in both lung regions from *CFTR*^*−/−*^ lambs, while secretory epithelial and basal cells are underrepresented in proximal lung, as are T cells and monocytes in distal lung. The imbalance in ciliated and basal cells was confirmed by immunohistochemistry. At 120 days of gestation, lymphoid cells are slightly more abundant in proximal and distal lung from *CFTR*^*−/−*^ animals compared to WT, consistent with the transient CF-associated inflammatory response in utero. At 80 days of gestation, T and B cells are underrepresented in both lung regions.

**Conclusions:**

The differences in epithelial cell abundance observed in the *CFTR*^*−/−*^ lambs at term may reflect sequelae from the loss of CFTR on lung development and differentiation in utero. These findings provide novel insights into the cellular mechanisms of pathology and may be relevant to the design of new therapeutic approaches for CF lung disease.

**Supplementary Information:**

The online version contains supplementary material available at 10.1186/s10020-025-01266-7.

## Background

The cellular architecture of the human lung is now mapped with remarkable precision, based upon multiple single cell RNA-seq analyses (Montoro et al. 2018; Plasschaert et al. 2018; Vieira Braga et al. 2019; Deprez et al. 2020; Travaglini et al. 2020; Sikkema et al. 2023) and the combined efforts of several international consortia including the Human Cell Atlas (https://www.humancellatlas.org), the Human Biomolecular Atlas program (https://hubmapconsortium.org) and LungMap (https://www.lungmap.net) among others (Regev et al. 2017; HubMAP 2019; Sun et al. 2022; Guo et al. 2023; Sikkema et al. 2023). These studies have identified marker gene signatures for rare or novel cell types and enhanced the understanding of lineage trajectories in the healthy lung. Moreover, the impact of disease states on lung biology is increasingly revealed by single cell studies that discover divergence from the normal atlas and cellularity, or alterations in gene expression patterns in specific cell types, for example in chronic obstructive pulmonary disease (COPD), asthma and cystic fibrosis (CF) (Schupp et al. 2020; Carraro et al. 2021; Sauler et al. 2022; Booth et al. 2023).

Key aspects of human lung development have also been the focus of single cell transcriptomics though are necessarily limited by the availability of human fetal tissues for research (Miller et al. 2020; He et al. 2022; Barnes et al. 2023). Similarly, the ability to investigate the early impact of lung disease in utero is greatly restricted. One common lung disease that likely initiates before birth is the inherited disorder CF, which is caused by mutations in the cystic fibrosis transmembrane conductance regulator (*CFTR*) gene. Newborns with CF generally have normal lung anatomy and physiology, though are susceptible to cycles of lung inflammation, infection and tissue remodeling that are characteristic features of the disease (Regamey et al. 2011; Byrnes et al. 2013; Giddings and Esther 2017; Deschamp et al. 2019). Moreover, 84% of babies with CF were reported to have cough symptoms within the first year of life (Goetz et al. 2019). The inability to accurately monitor the human CF lung disease process before birth at the cellular level may be partially addressed by utilizing large animal models of the disease, which have similar lung anatomy, physiology and developmental trajectories. Both the pig (Rogers et al. 2008a, 2008b) and the sheep (Fan et al. 2018; Viotti Perisse et al. 2021) models of CF meet these criteria, though interestingly the gross impact of loss of CFTR function on the developing lung is not identical in the two species (Meyerholz et al. 2018; Van Wettere et al. 2022). Single cell RNA-sequencing (scRNA-seq) allows a more detailed analysis of cell identities and trajectories in the developing lung and how these features may be altered by CF disease processes. A recent scRNA-seq analysis of the neonatal pig lung at birth reported a minimal impact of loss of CFTR on the transcriptome of large and small airways at birth (Thurman et al. 2022). However, this study focused on micro-dissected airway tissue, with large airway epithelium obtained by tracheal scraping and small airways isolated intact following removal of vasculature and parenchymal tissue. Equivalent analyses have not yet been done on the CF ferret models, which have an accelerated postnatal lung phenotype compared to human, rapidly developing lung infections in the first week of life (Sun et al. 2010).

Here, we describe global scRNA-seq analysis of proximal and distal lung regions from the CF sheep model and age-matched wild-type sheep, without prior isolation of any specific cell type or prediction of likely sites of disease. Our definition of proximal and distal lung tissue is a regional one, rather than referring to airway diameter. Moreover, we collect tissues at 80 days and 120 days of sheep gestation, broadly equivalent to ~ 21 weeks and > 30 weeks in human in addition to harvesting at natural term (147 days). Our results indicate that distributions of cell populations in the fetal sheep lung are impacted by loss of CFTR, albeit differently at 120 days gestation and at term. The observations at term may contribute to understanding the susceptibility of CF newborns to lung infection.

## Methods

The materials and methods for this study were as described in our previous work (Van Wettere et al. 2022; Kerschner et al. 2023; Leir et al. 2024), but are briefly reiterated here, with detailed methods provided in the Supplementary Methods file.

### Sex as a biological variable

Our study examined male and female animals where possible since all cloned animals used here are male. Results are combined and sex was not considered as a biological variable.

### Animals

American Romney breed of domestic sheep (*Ovis aries*) was used in this study. All animal studies were approved and monitored by the Institutional Animal Care and Use Committee (IACUC) at Utah State University (IACUC protocol # 10089) and conformed to the National Institute of Health guidelines. WT sheep were bred according to standard protocols. *CFTR*^*−/−*^ sheep pregnancies were generated by somatic cell nuclear transfer (SCNT) or by natural breeding as shown in Table 1.

**Table 1:** Samples for scRNA-seq analysis

Age	Distal/ Proximal	scRNA-seq ID/(cell #)	Animal #	Genotype	Sex	Cloned/Natural
80 d	Proximal	AXH035 (3163)	SF1501-1	WT	M	natural
80 d	Proximal	AXH064 (2169)	SFWT194-2	WT	M	natural
80 d	Proximal	AXH063 (411)	SFWT194-1	*CFTR*^*+/−*^	M	natural
80 d	Proximal	AXH065 (1782)	SFWT194-3	*CFTR*^*+/−*^	M	natural
80 d	Proximal	AXH058 (2941)	SFY1722-1	*CFTR *^*−/−*^	M	cloned
80 d	Proximal	AXH119 (3679)	SFP2050-1	*CFTR *^*−/−*^	M	cloned
80 d	Proximal	AXH120 (8681)	SFP2050-2	*CFTR *^*−/−*^	M	cloned
80 d	Distal	AXH034 (3532)	SF1501-1	WT	M	natural
80 d	Distal	AXH061 (1984)	SFWT194-2	WT	M	natural
80 d	Distal	AXH060 (2461)	SFWT194-1	*CFTR*^*+/−*^	M	natural
80 d	Distal	AXH062 (590)	SFWT194-3	*CFTR*^*+/−*^	M	natural
80 d	Distal	AXH057 (2107)	SFY1722-1	*CFTR *^*−/−*^	M	cloned
80 d	Distal	AXH117 (3346)	SFP2050-1	*CFTR *^*−/−*^	M	cloned
80 d	Distal	AXH118 (3123)	SFP2050-2	*CFTR *^*−/−*^	M	cloned
120 d	Proximal	AXH043 (3575)	SF1301-1	WT	M	natural
120 d	Proximal	AXH044 (4104)	SF1301-2	WT	F	natural
120 d	Proximal	AXH071 (11,031)	SFWT1601-2	WT	M	natural
120 d	Proximal	AXH072 (9810)	SFWT1601-3	WT	M	natural
120 d	Proximal	AXH077 (7172)	SFY1705 A*	*CFTR *^*−/−*^	M	cloned
120 d	Proximal	AXH078 (7553)	SFY1705B*	*CFTR *^*−/−*^	M	cloned
120 d	Proximal	AXH125 (4458)	SFY1905-1	*CFTR *^*−/−*^	M	cloned
120 d	Proximal	AXH126 (3864)	SFY1905-2	*CFTR *^*−/−*^	M	cloned
120 d	Proximal	AXH129 (3629)	SFP2077	*CFTR *^*−/−*^	M	cloned
120 d	Proximal	AXH134 (4537)	SFCF2502-1	*CFTR *^*−/−*^	F	natural
120 d	Proximal	AXH135 (3895)	SFCF2502-2	*CFTR *^*−/−*^	F	natural
120 d	Distal	AXH041 (3209)	SF1301-1	WT	M	natural
120 d	Distal	AXH042 (3052)	SF1301-2	WT	F	natural
120 d	Distal	AXH069 (8983)	SFWT1601-2	WT	M	natural
120 d	Distal	AXH070 (7754)	SFWT1601-3	WT	M	natural
120 d	Distal	AXH075 (10,137)	SFY1705 A*	*CFTR *^*−/−*^	M	cloned
120 d	Distal	AXH076 (9983)	SFY1705B*	*CFTR *^*−/−*^	M	cloned
120 d	Distal	AXH123 (3232)	SFY1905-1	*CFTR *^*−/−*^	M	cloned
120 d	Distal	AXH124 (3516)	SFY1905-2	*CFTR *^*−/−*^	M	cloned
120 d	Distal	AXH128 (3860)	SFP2077	*CFTR *^*−/−*^	M	cloned
120 d	Distal	AXH132 (5424)	SFCF2502-1	*CFTR *^*−/−*^	F	natural
120 d	Distal	AXH133 (5829)	SFCF2502-2	*CFTR *^*−/−*^	F	natural
term 72 h	Proximal	AXH021 (2690)	WT2004	WT	M	natural
term 62 h	Proximal	AXH141 (4706)	SFWT1615	WT	M	natural
term 14 h	Proximal	AXH047 (6710)	2298	WT	F	natural
term 6 h	Proximal	AXH038 (2761)	SF173-1	WT	M	natural
term 5.5 h	Proximal	AXH138 (4770)	SFWT2281	WT	F	natural
term 60 h	Proximal	AXH086 (3051)	CF2103	*CFTR *^*−/−*^	F	natural
term 18 h	Proximal	AXH053 (5978)	CF2504	*CFTR *^*−/−*^	F	natural
term ~ 16 h	Proximal	AXH111 (4087)	SFCF2114	*CFTR *^*−/−*^	F	natural
term 9 h	Proximal	AXH146 (5142)	SFP2058 A*	*CFTR *^*−/−*^	M	cloned
term 9 h	Proximal	AXH147 (6450)	SFP2058 B*	*CFTR *^*−/−*^	M	cloned
term 72 h	Distal	AXH022 (2273)	WT2004	WT	M	natural
term 62 h	Distal	AXH140 (4296)	SFWT1615	WT	M	natural
term ~ 16 h	Distal	AXH089 (11,932)	SFWT2011-1	WT	M	natural
term 14 h	Distal	AXH046 (10,012)	2298	WT	F	natural
term 6 h	Distal	AXH037 (2900)	SF173-1	WT	M	natural
term 5.5 h	Distal	AXH137 (4172)	SFWT2281	WT	F	natural
term 60 h	Distal	AXH085 (4643)	CF2103	*CFTR *^*−/−*^	F	natural
term 18 h	Distal	AXH050 (12,844)	CF2503	*CFTR *^*−/−*^	M	natural
term 18 h	Distal	AXH051 (9068)	CF2504	*CFTR *^*−/−*^	F	natural
term ~ 16 h	Distal	AXH110 (3623)	SFCF2114	*CFTR *^*−/−*^	F	natural
term 9 h	Distal	AXH144 (5288)	SFP2058 A*	*CFTR *^*−/−*^	M	cloned
term 9 h	Distal	AXH145 (5328)	SFP2058 B*	*CFTR *^*−/−*^	M	cloned

Key: * denotes 2 separate samples from one animal

### Histopathologic analysis and immunochemistry

A necropsy was performed on all fetuses collected, to examine for gross lesions and the findings were documented as described previously (Van Wettere et al. 2022). Lung tissue samples were collected and fixed in 10% neutral buffered formalin for histology. For immunochemistry, primary antibodies FOXJ1 (Abcam ab235445), KRT5 (Invitrogen MA5-16372) and SCGB3A2 (Abcam ab181853) and secondary antibody Alexa Fluor 488 AffiniPure Goat Anti-Rabbit IgG (H + L) (Jackson ImmunoResearch) were used. Images were captured by the Lionheart FX Automated Microscope (BioTek) and cell quantification performed using Gen5 software (BioTek). Briefly, at least five bronchioles were randomly selected from each sample, the epithelium was delineated manually and then cells within the delineation were counted automatically. The percentages of FOXJ1-positive apical ciliated epithelial cells and KRT5-positive basal cells were calculated by dividing the number of FOXJ1- or KRT5-positive cells by the total number of DAPI-positive apical or basal cells, respectively. Proximal lung sections from three different term WT and CF animals were analyzed. The results represent a total of > 1100 epithelial cells analyzed for FOXJ1 and > 800 epithelial cells analyzed for KRT5.

### Single-cell RNA sequencing (scRNA-seq)

i) *Isolation of single cells from tissues. *Tissues for scRNA-seq were from WT animals at 80-, 120- and 147 days (term) gestation, cloned *CFTR*^*−/−*^ animals at 80-, 120-days and term, and naturally bred *CFTR*^*−/−*^ lambs at 120-days and term. Proximal and distal lung regions for single cells isolation are shown in Fig. S1 and detailed protocols are in the Supplementary Methods.
ii) *Single-cell RNA-sequencing and analysis*
  i) Approximately 3000-5000 cells were used for scRNA-seq using the 10X Genomics Chromium Single Cell 3’ Reagent Kit v3, or v3.1. The libraries were sequenced on a NovaSeq 6000 machine. Reads were aligned to the Oar_v4.0/oviAri4 (Texel) genome using Cell Ranger 3.1.0. Cells were filtered for quality using cuts for standard metrics: library size, number of detected genes and mitochondrial read percentage. Ribosomal protein genes were also filtered out. The resulting objects were normalized and batch-corrected using the Seurat R package (Hao et al. 2021), version 5.1.0, followed by clustering and UMAP (Uniform Manifold Approximation and Projection) dimensionality reduction. Seurat was also used to find cluster markers by performing differential gene expression analysis between clusters using the receiver operating characteristic (ROC) method. To compare cell proportions between *CFTR*^*−/−*^ and WT in each cluster, a Monte-Carlo/permutation test was performed using the scProportionTest R package (https://github.com/rpolicastro/scProportionTest) (Miller et al. 2021).
  ii) *Pseudotime analysis* was performed in R (version 4.4.1) using the monocle3 package (version 1.4.17) (https://cole-trapnell-lab.github.io/monocle3). Twenty seven wild type (WT) (4 distal and 4 proximal at 80-days, 4 distal and 4 proximal at 120-days of gestation and 6 distal and 5 proximal at term) and thirty three *CFTR*^*−/−*^ (4 distal and 4 proximal at 80-days, 7 distal and 7 proximal at 120-days of gestation and 6 distal and 5 proximal at term) samples were separately run through the same pipeline.

#### Statistics

Statistical methods are built into the single-cell analysis pipelines as described above. Statistical tests for other assays are described in the Figures and Figure Legends.

#### Study approval

All animal studies were approved and monitored by the Institutional Animal Care and Use Committee (IACUC) at Utah State University (IACUC protocol # 10089) and conformed to the National Institute of Health guidelines.

### Data availability

All sequencing data from this project are deposited at GEO: GSE281174.

## Results

For scRNA-seq experiments we collected lung tissue from proximal and distal lung (regions defined in Fig. S1) at 80 days and 120 days gestation and at term from multiple animals as described in Table 1. At 80 days tissues were collected from four non-CF animals (2 WT and 2 heterozygote *CFTR*^*+/−*^) and three CF animals (*CFTR*^*−/−*^). A total of 8,567 distal lung cells and 7,525 proximal non-CF cells, and 8,585 distal lung cells and 15,301 proximal lung from *CFTR*^*−/−*^ animals were analyzed. At 120 days tissues were from 4 non-CF (WT) animals, 6 cloned *CFTR*^*−/−*^ animals and 2 *CFTR*^*−/−*^ produced by natural breeding. A total of 22,998 distal and 51,518 proximal cells from WT and 41,918 distal and 35,088 proximal cells from *CFTR*^*−/−*^ animals were analyzed. At term (specific recorded times between 5.5 h and 72 h post-partum) tissues were from 6 WT animals and 5 *CFTR*^*−/−*^ animals, 4 of these were produced by natural breeding and one by cloning. A total of 35,585 distal lung cells and 21,637 proximal lung cells from WT and 40,794 distal lung cells and 24,708 proximal lung cells from *CFTR*^*−/−*^ animals were analyzed. In all cases the cell numbers reflect the post processing data generated by the 10X Genomics Cell Ranger v 3.1.0 pipeline. In total across the sheep lung developmental time course, we have captured 314,224 cells for analysis. In the first instance we processed the output from Cell Ranger for WT and *CFTR*^*−/−*^ animals separately to ensure that individual samples were not outliers, for example if only contributing to a subset of clusters with a high abundance of cells in those clusters. Four term samples were discarded as a result and are not included in Table 1. Next, we merged the data from the WT and *CFTR*^*−/−*^ animals at each time point, keeping the proximal and distal tissues separate. The results are presented in reverse chronological order as cluster identities were clearer at term than at earlier time points.

### Overrepresentation of ciliated cells and underrepresentation of secretory epithelial cells and basal cells in proximal lung of* CFTR*^*−/− *^lambs at term

**Fig. 1:**
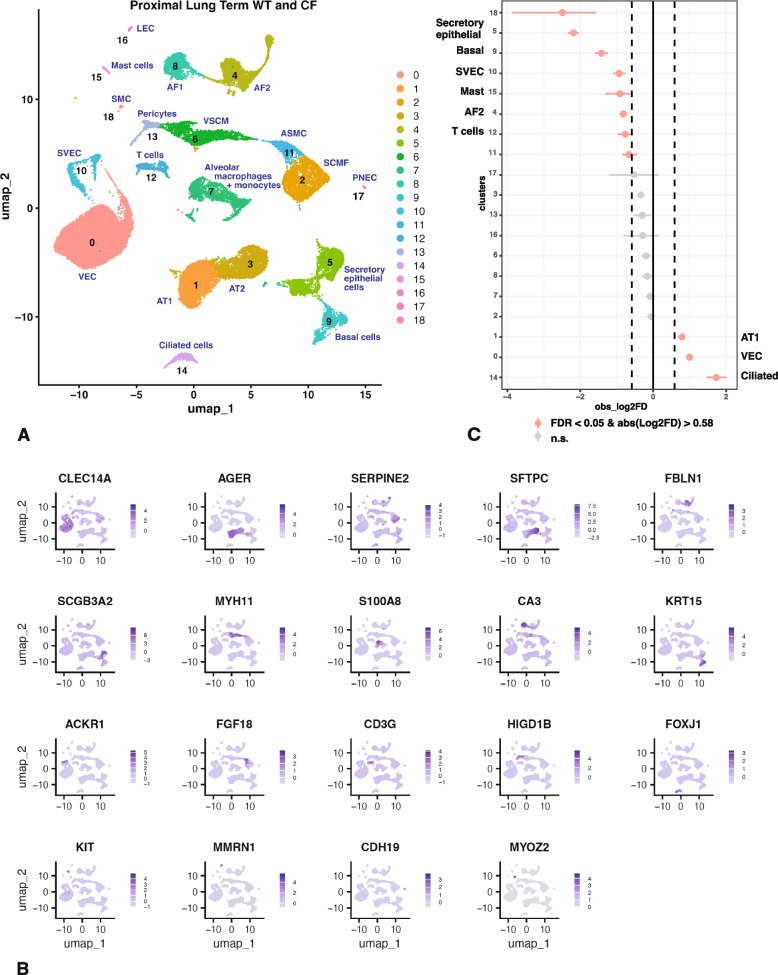
Single cell RNA-seq defines cell types in term WT and *CFTR*^*−/−*^ sheep proximal lung and shows overrepresentation of FOXJ1+ ciliated cells and underrepresentation of secretory epithelial cells and basal cells in *CFTR*^*−/−*^ tissues. **A** UMAP plot of merged data from 5 WT and 5 *CFTR*^*−/−*^ donors identifies 19 clusters by differential gene expression profiles, each named by cell type. Abbreviations: VEC, vascular endothelial cell; AT1/2, alveolar type 1/2; SCMF, secondary crest myofibroblast; AF1/2, alveolar fibroblasts (1/2); VSMC, vascular smooth muscle cells; SVEC, systemic venous endothelial cells; ASMC, airway smooth muscle cells; LEC, lymphatic endothelial cells; PNEC, neuroendocrine cells; SMC smooth muscle cells. **B** Feature plots show expression of one marker for each cell type/cluster identified in (**A**) and described in the text: 0, *CLEC14A*; 1, *AGER*; 2, *SERPINE2*; 3, *SFTPC*; 4, *FBLN1*; 5, *SCGB3A2*; 6, *MYH11*; 7, *S100A8*; 8, *CA3*; 9, *KRT15*; 10, *ACKR1*; 11, *FGF18*; 12, *CD3G*; 13, *HIGD1B*; 14, *FOXJ1*; 15, *KIT*; 16, *MMRN1*; 17, *CDH19*; 18, *MYOZ2*. **C** Single cell proportions test comparing *CFTR*^*−/−*^ to WT shows significant overrepresentation of ciliated cells (14) and underrepresentation of secretory epithelial cells (5) and basal cells (9) in *CFTR*^*−/−*^ tissues

The data from 6 WT animals and 5 *CFTR*^*−/−*^ animals at term were merged keeping the proximal and distal tissues separate and not considering precise hours after birth as a variable. Using a resolution of 0.2 in the Seurat pipeline with the filtering parameters stated in the methods section we identified 19 cell clusters in the merged WT and *CFTR*^*−/−*^ proximal lung samples at term. Cluster resolution by identity is shown in Fig. 1A and by sample in Fig. S2. Clusters were assigned a cellular identity by inspection of the marker gene lists shown in Table S1 A and correlation with known gene expression patterns in the human proximal lung. The feature plots in Fig. 1B show one marker for each cluster and the dot plot in Fig. S3 A further confirms cluster identity. For additional validation of cluster assignment we referred to LungMAP (Guo et al. 2023) as a valuable resource for cell identity in the human and mouse lungs, though some markers were not available to us due to the incomplete annotation of the sheep genome.

i) *Cluster identity by differential gene expression profiles in proximal lung at term.*

The most abundant cluster 0 represents endothelial cells, most probably vascular endothelial cells (VEC) as identified by cadherin 5 (*CDH5*), C-type lectin domain containing 14A (*CLEC14A*), SRY-box transcription factor 17 (*SOX17*), endomucin (*EMCN*) and platelet and endothelial cell adhesion molecule 1 (*PECAM1*) on the marker gene list and the absence of homeobox D9 (*HOXD9*) and pentraxin 3 (*PTX3*). Cluster 1 are alveolar type 1 (AT1) cells with the characteristic marker genes of advanced glycosylation end-product specific receptor (*AGER*), HOP homeobox (*HOPX*), rhotekin 2 (*RTKN2*), epithelial membrane protein 2 (*EMP2*), claudin 18 (*CLDN18*) and the cut like homeobox 1 (*CUX1*) transcription factor among others, and without ATPase H + transporting V1 subunit C2 (*ATP6V1C2*) and ETS variant transcription factor 5 (*ETV5*). The neighboring cluster 3 in UMAP space contains alveolar type 2 (AT2) cells which have multiple genes encoding surfactant proteins on the marker gene list, such as *SFTPC*, *SFTPB* and *SFTPD*. The marker gene list for Cluster 2 cells is comparatively short, and it is difficult to unequivocally assign an identity, though they are clearly mesenchymal cells, with decorin (*DCN*), collagen type III alpha 1 chain (*COL3A1*) and collagen type VI alpha 1 chain (*COL6A1*) on the list. The presence of transforming growth factor beta induced (*TGFBI*), syndecan 2 (*SDC2*) and platelet derived growth factor receptor alpha (*PDGFRA*) (Li et al. 2019) as marker genes, and fibroblast growth factor 18 (*FGF18*) expression (though not on the marker gene list) suggests they may be secondary crest myofibroblast cells, which will be described more fully in the distal lung section. Clusters 4 and 8 are likely alveolar fibroblasts, AF2 and AF1 cells respectively. Cluster 4 markers include microfibril associated protein 5 (*MFAP5*), peptidase inhibitor 16 (*PI16*), osteoglycin (*OGN*), coiled-coil domain containing 80 (*CCDC80*) and multiple collagen genes, among them *COL14A1*, *COL3A1*, *COL1A2* and *COL5A1*. Cluster 8 markers indicate AF1 cells, with transcription factor 21 (*TCF21*), collagen type XIII alpha 1 chain (*COL13A1)*, carbonic anhydrase 3 (*CA3*) and glypican 3 (*GPC3*) on the marker gene list. Also close to each other in UMAP space are clusters 5 and 9, which are likely secretory epithelial cells and basal cells respectively. Markers for cluster 5 include secretoglobin family member 2 A member 2 (*SCGB2A2*) and 3 A member 2 (*SCGB3A2*), trefoil factor 3 (*TFF3*), WAP four-disulphide core domain 2 (*WFDC2*) and the transcription factors ETS homologous factor (*EHF*) and krüppel-like factor 5 (*KLF5*), both of which we showed previously to have pivotal roles in primary human airway epithelial cells (Fossum et al. 2014, 2017; Paranjapye et al. 2021, 2022b). Markers for cluster 9 include keratin 5, 14 and 15 (*KRT5*, *KRT14*, *KRT15*) and tumor protein P63 (*TP63*). Clusters 6 and 11 are other mesenchymal cell types with cluster 6 likely to be vascular smooth muscle cells (VSMC) with musculoskeletal, embryonic nuclear protein 1 (*MUSTN1*), phospholamban (*PLN*), calponin 1 (*CNN1*) and integrin subunit alpha 7 (*ITGA7*) as marker genes. Cluster 11 has features of airway smooth muscle cells (ASMC) with transgelin (*TAGLN*), desmin (*DES*), actin alpha 2, smooth muscle (*ACTA2*), actin gamma 2, smooth muscle (*ACTG2*), myosin light chain kinase (*MYLK*), myosin heavy chain 11 (*MYH11*) and tropomyosin 2 (*TPM2*) on the marker gene list. However, these cells, which are not distinguished from cluster 2 at a resolution of 0.1, and have the highest expression of FGF18, which might also be indicative of secondary crest myofibroblast cells (SCMF) cells. Clusters 7, 12 and 15 are all immune cell types with distinct identities. The presence of S100 calcium binding protein A8 and A9 (*S100A8* and *S100A9*) and colony stimulating factor 1 receptor (*CSF1R*) in the marker gene list for cluster 7 is consistent with monocytes and alveolar macrophages. The distribution of *S100A8* and *CSF1R* expression within cluster 7 suggests these two populations are different (*CSF1R* is not shown in Fig. 1B) but are not sufficiently divergent to generate separate clusters at this resolution. Cluster 12 are T cells, which express high levels of CD3 epsilon and CD3 gamma subunits of T-cell receptor complex (*CD3E*, *CD3G*), protein tyrosine phosphatase receptor type C associated protein (*PTPRCAP*) and protein tyrosine phosphatase receptor type C (*PTPRC*). Both T cell receptor delta locus (*TRD*) and *CD7* are markers for cluster 12, consistent with gamma delta T cells. Cluster 15 are mast cells with KIT proto-oncogene, receptor tyrosine kinase (*KIT*), Fc epsilon receptor Ig (*FCER1G*), membrane spanning 4-domains A2 (*MS4A2*), leukotriene C4 synthase (*LTC4S*) and tryptase beta-2 (*TPSB2*) on the marker gene list. Cluster 10 cells are endothelial cells, most probably systemic venous endothelial cells (SVEC) with atypical chemokine receptor 1 (duffy blood group) (*ACKR1*), plasmalemma vesicle associated protein (*PLVAP*), Von Willebrand factor (*VWF*) and *PECAM1* on the marker gene list, however they may also encompass arterial endothelial cells (AEC). Cluster 13 show markers of pericytes, including HIG1 hypoxia inducible domain family member 1B (*HIGD1B*), family with sequence similarity 162 member B (*FAM162B*), cytochrome C oxidase subunit 42(*COX4I2*) and NDUFA4 mitochondrial complex associated like 2 (*NDUFA4L*). Cluster 14 are forkhead box J1 (*FOXJ1*)-expressing ciliated cells with tetraspanin 1 (*TSPAN1*), leucine rich repeats and IQ motif containing 1 (*LRRIQ1*) and calcyphosine like (*CAPSL*) in addition to FOXJ1 on the marker gene list. Cluster 16 are lymphatic endothelial cells (LEC) marked by high expression of multimerin 1 (*MMRN1*) and prospero homeobox1 (*PROX1*). The identities of clusters 17 and 18, which contain very few cells are not definitive though pulmonary neuroendocrine cells (PNEC) may contribute to cluster 17, marked by neurexin (*NRXN1*) though few other marker genes for these cells are annotated in the sheep genome. Cluster 18 may include smooth muscle cells (SMC) as myozenin 2 (*MYOZ2*) and myosin light chain 7 (*MYL7*) are dominant marker genes, though features of other cell types are also evident.

ii) *Cluster abundance in proximal lung of WT and CFTR* ^*−/−*^ *lambs at term*

Having identified the majority of cell clusters in the proximal lung tissue at term, we next compared the abundance of each cluster in WT and *CFTR*^*−/−*^ tissues using a single cell proportions test with significance defined as a false discovery rate (FDR) of < 0.05 and an absolute log2 fold difference (FD) > 0.58 (Fig. 1C). Cell numbers are shown in Table S1. The most overrepresented cluster in the *CFTR*^*−/−*^ proximal lung tissue compared to WT are FOXJ1-expressing ciliated cells, with vascular endothelial cells (cluster 1, VEC) and alveolar type 1 cells (cluster 1, AT1) just reaching statistical significance. In contrast secretory epithelial cells (cluster 5), and basal cells (cluster 9) are the most significantly underrepresented in the *CFTR*^*−/−*^ proximal lung tissue compared to WT. Several other clusters just reach statistical significance as depleted in the *CFTR*^*−/−*^ tissues including SVEC (cluster 10), mast cells/basophils (cluster 15), AF2 cells (cluster 4), T cells (cluster 12) and ASMC/SCMF cells (cluster 11) though these minor statistical differences may not be biologically significant.

We reclustered cluster 5 to determine whether the underrepresentation of secretory epithelial cells in *CFTR*^*−/−*^ animals in proximal lung at term related to a specific cell type (Fig. S4A; Table S2A). The UMAP by cluster identity in Fig. S4A shows 6 distinct cell types within cluster 5. The most abundant cell type (0) are secretory epithelial cells marked by *SCGB3A2*, *EHF* and *KLF5*, cluster 1 is marked by multiple surfactant genes suggestive of AT2 cells but not *SFTPC,* and also has the AT1 cell marker *AGER* so we define these cells as AT0/1/2, cluster 2 have characteristics of basal cells (*KRT15*) and mucus cells (BPI fold containing family B member 1, *BPIFB1*) and may represent a transitional state. Cluster 3 are likely goblet cells marked by *TFF3* and also the ovine homologue of *MUC5B* (LOC101105728), (of note, the ovine homologue of *MUC5AC* (LOC106991828, LOC106991829) was not expressed in the lung). Cluster 4 are mesenchymal cells and cluster 5 are adipocytes, which are contributed by only one tissue donor and may be a contaminating artifact. When comparing cluster abundance in the proportions test (Fig. S4B) the secretory epithelial cells in cluster 0 are significantly underrepresented in the *CFTR*^*−/−*^ samples while AT0/1/2 cells are overrepresented.

Lastly, we were interested to know whether the inclusion of proximal lung tissue from a cloned *CFTR*^*−/−*^ term lamb (2 separate cell samples) with the three naturally bred term animals was influencing the cell proportions test results. To address this point, we excluded the data from the cloned animal (samples AXH146 and AXH147) from the Seurat analysis and showed that FOXJ1-ciliated cells and AT1 cells were still the most overrepresented clusters in the non-cloned *CFTR*^*−/−*^ animals and the most significantly underrepresented cluster in the non-cloned animals alone was secretory epithelial cells (data not shown).

### Overrepresentation of ciliated cells and underrepresentation of immune cells in distal lung of *CFTR*^*−/− *^lambs at term

**Fig. 2:**
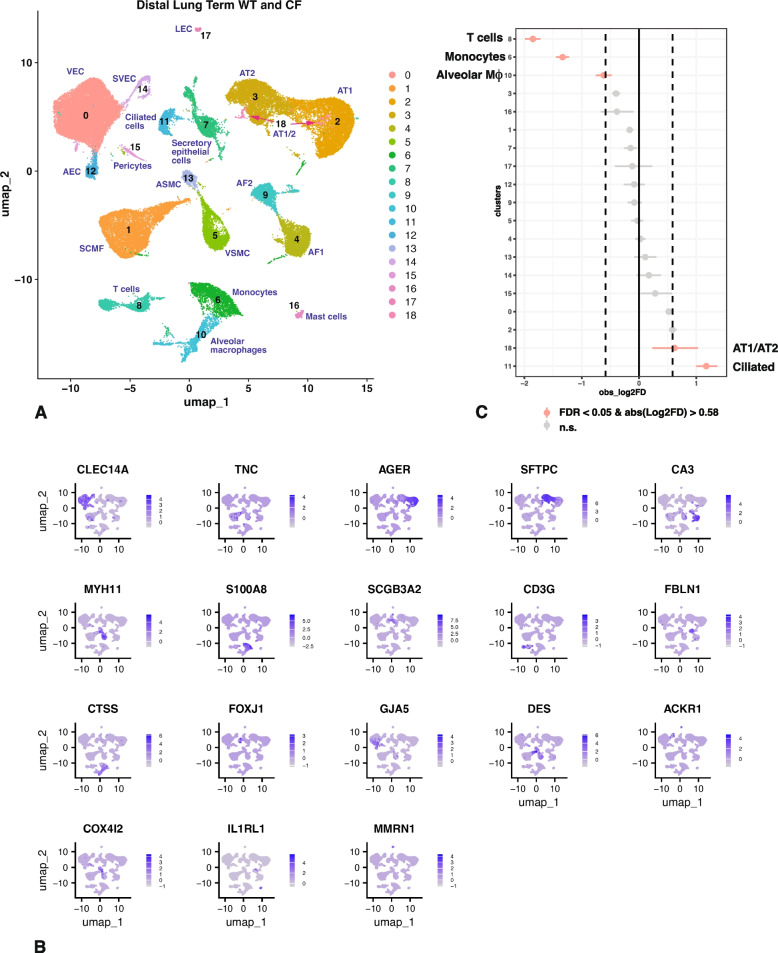
Single cell RNA-seq defines cell types in term WT and *CFTR*^*−/−*^ sheep distal lung and shows overrepresentation of FOXJ1+ ciliated cells and underrepresentation of T-cells and monocytes in *CFTR*^*−/−*^ tissues. **A** UMAP plot of merged data from 6 WT and 6 *CFTR*^*−/−*^ donors identifies 19 clusters by differential gene expression profiles, each named by cell type. Abbreviations are defined in the legend to Fig. 1, except AEC, arterial endothelial cells. **B** Feature plots show expression of one marker for each cell type/cluster identified in **A** and described in the text: 0, *CLEC14A*; 1, *TNC*; 2, *AGER*; 3, *SFTPC*; 4, *CA3*; 5, *MYH11*; 6, *S100A8*; 7, *SCGB3A2*; 8, *CD3G*; 9, *FBLN1*; 10, *CTSS*; 11, *FOXJ1*; 12, *GJA5*; 13, *DES*; 14, *ACKR1*; 15, *COX412*; 16, *IL1RL1*; 17, *MMRN1*; 18, has markers of both clusters 2 and 3. **C** Single cell proportions test comparing *CFTR*^*−/−*^ to WT shows significant overrepresentation of ciliated cells (11) and underrepresentation of T cells (8) and monocytes (6) in *CFTR*^*−/−*^ tissues

As for proximal lung we used a resolution of 0.2 in the Seurat pipeline with the filtering parameters stated in the methods section and identified 19 cell clusters in the merged WT and *CFTR*^*−/−*^ distal lung samples at term. Cluster resolution by identity is shown in Fig. 2A and by sample in Fig. S2B. Clusters were assigned a cellular identity by inspection of the marker gene lists shown in Table S3 and correlation with known gene expression patterns in the human proximal lung. Additional validation of cluster assignment was again from LungMAP (Guo et al. 2023).

i) *Cluster identity by differential gene expression profiles in distal lung at term.*

Clusters are shown by feature plot in Fig. 2B and by dot plot in Fig. S3B. The most abundant cluster 0 represents VEC, with *CDH5, CLEC14A*, *PECAM1, SOX1*, *EMCN,* and receptor activity modifying protein 2 (*RAMP2*) on the marker list, but as expected for VEC not *PTX3*. The next largest cell cluster 1 are a mesenchymal cell population that are likely SCMF cells. SCMF cells are a transient lineage that drives alveolar septa formation during alveogenesis, a process that commences in the lung in utero (after 120 days in the sheep lung) and continues after birth (Schittny 2017). Among key marker genes for SCMF cells that are seen in cluster 1 are *FGF18*, *TGFBI*, tenascin C (*TNC*), *COL3A1* and collagen type XII alpha 1 chain (*COL12A1*), while negative markers for these cells including G0/G1 switch 2 (*G0S2*), ATPase Na^+^/K^+^ transporting subunit alpha 1 (*ATP1A1*), plexin domain containing 2 (*PLXDC2*) and *TCF21* are not on the marker gene list. Cluster 2 and cluster 3 are AT1 and AT2 cells respectively. Markers for cluster 2 include *AGER*, *HOPX, RTKN2*, *EMP2*, *CLDN18*, LIM domain 7 (*LMO7*) and KRT7, while negative markers *ATP6V1C2* and acyl-coA synthetase long chain family member 4 (*ACSL4*) are not on the list. Cluster 3 markers include lysosomal associated membrane protein 3 (*LAMP3*), multiple surfactant genes such as *SFTPC*, *SFTPB* and *SFTPD*, solute carrier family 34 member 2 (*SLC34A2*) which encodes a pH-sensitive and sodium-dependent phosphate transporter, which is mutated in pulmonary alveolar microlithiasis, a disease characterized by calcium phosphate deposits in the lung. Other markers include surfactant associated 2 (*SFTA2*) and C-X-C motif chemokine ligand 17 (*CXCL17*), while the negative marker aquaporin 5 (*AQP5*) is excluded. Of interest are cluster 18, which are embedded in the same UMAP space as AT1 and AT2 cells and may be transitional cells expressing some markers of both cell types, which are hypothesized to have unique roles in lung development and disease (Sucre et al. 2023; Wang et al. 2023). Clusters 4 and 9 appear to be AF1 and AF2 cells respectively with the marker gene list for cluster 4 including *CA3*, *TCF21*, *COL13A1*, LBH regulator of WNT signaling pathway (*LBH*), *ITGA8* and *CDH11*, while *MYH11* and *TGFBI* are absent. Among markers of cluster 9 are *CCDC80*, *MFAP5*, *DCN*, *COL1A1*, *COL1A2*, *COL3A1* and procollagen C-endopeptidase enhancer (*PCOLCE*), while the negative signature T-box transcription factor 5 (*TBX5*) is absent. Clusters 5 and 13, which are also close in UMAP space have marker genes consistent with VSMC and ASMC respectively. Marker genes of cluster 5 include *CNN1*, *ITGA7*, *MYH11, TPM1* and myosin light chain 6 (*MYL6*), while *TBX5* is absent. Meanwhile, cluster 13 markers include *DES*, *ACTA2*, *TPM2* and *TAGLN*. Clusters 6, 8 and 10 are all immune cell types that are somewhat challenging to identify definitively in the developing sheep (Leir et al. 2024). Cluster 6 are indicative of myeloid cells, likely monocytes *S100A8*, *S100A9*, cytochrome B-245 beta chain (*CYBB*) and interleukin 1 beta (*IL1B*) on the marker gene list. Cluster 8 are T cells, with *CD3E*, and *CD3G* close to the top of the marker gene list, which also shows *CD3D*, dual specificity phosphatase 2 (*DUSP2*) and the interleukin 7 receptor (*IL7R*). As for cluster 12 in the proximal term lung, both *TRD* and *CD7* are markers 8, consistent with gamma delta T cells. Cluster 10 cells have a gene expression profile indicative of alveolar macrophages, with arachidonate 5-lipoxygenase activating protein (*ALOX5AP*), the scavenger receptor family member *CD68*, *FCER1G*, the Spi-1 proto-oncogene (*SPI1*) an ETS domain transcription factor that regulates myeloid and B-lymphoid development (Pham et al. 2013) and cathepsin S (*CTSS*) all on the marker gene list. Cluster 7 are secretory epithelial cells marked by *TFF3*, *SCGB3A2*, *KLF5* and *EHF*. The identity of cluster 11 as multiciliated cells (reviewed in (Davis and Wypych 2021)) is clear with the transcription factor *FOXJ1* on the marker gene list together with *TSPAN1*, radial spoke head component 1 (*RSPH1*), dynein light chain roadblock-type 2 (*DYNLRB2*), cilia and flagella associated protein 144 (*CFAP144*) and tubulin beta 4B class IVb (*TUBB4B*). The negative signatures AQP5, solute carrier family 39 member 8 (*SLC39A8*) and ETV5 were on the marker gene lists for other clusters but not cluster 11. Clusters 12 and 14 are both subtypes of endothelial cells likely AEC and SVEC respectively, and both lie close to VEC (cluster 0) in UMAP space. The marker gene list for cluster 12 features gap junction protein alpha 5 (*GJA5*), protein tyrosine phosphatase receptor type B (*PTPRB*), Hes related family BHLH transcription factor with YRPW motif 1 (*HEY1*), notch receptor 1 (*NOTCH1*), *CXCL12* and BMX non-receptor tyrosine kinase (*BMX*), while that for cluster 14 includes *ACKR1* and *PLVAP* in addition to other common endothelial cell markers. Cluster 15 appear to be pericytes, with *FAM162B, HIGD1B* and *COX4I2* as diagnostic marker genes, cluster 16 are dendritic cells, with *FCER1A*, *RNASE4* and *S100A10* as marker genes. Lastly cluster 17 are LEC cells marked by high expression of *MMRN1* and *PROX1*.

ii) *Cluster abundance in distal lung of WT and* *CFTR* ^*-/-*^ *lambs at term.*

Next, having identified the majority of cell clusters in the distal lung tissue at term, we compared the abundance of each cluster in WT and *CFTR*^*−/−*^ tissues using a single cell proportions test with significance defined as FDR < 0.05 and an absolute log2 FD > 0.58 (Fig. 2C). As we observed in the *CFTR*^*−/−*^ proximal lung tissue, the most overrepresented cell type in the *CFTR*^*−/−*^ distal lung tissue compared to WT are FOXJ1-expressing ciliated cells in cluster 11. Transitional AT1/AT2 cells (cluster 18) also just reach statistical significance for increased abundance, thought the error bar on this small cluster is greater. However, unlike in the proximal lung where secretory cells and basal cells are underrepresented in the *CFTR*^*−/−*^ tissues, in the distal lung the most significantly underrepresented cells are immune cells, specifically T cells (cluster 8) and monocytes (cluster 6) with alveolar macrophages (cluster 10) also just reaching statistical significance.

We reclustered cluster 7 (secretory epithelial cells) to refine the cellular identities (Fig. S4 C; Table S2B). The UMAP by cluster identity in Fig. S4C shows 8 distinct cell types within cluster 7. The most abundant cell type (0) are AT0/1/2, (based on our definition of cluster 1, while reclustering term proximal lung cluster 5 above), cluster 1 are basal cells, cluster 2 are rapidly dividing cells, secretory epithelial/club cells are in cluster 3, while cluster 4, 5 and 6 are likely macrophages, VEC, and stromal cells respectively. Cluster 7 are contaminating erythrocytes. The proportions test (Fig S4D) suggests that there is no biological difference between the abundance of cell types between *CFTR*^*−/−*^ and WT tissues in this cellular compartment although secretory epithelial/club cells and macrophages just reach statistical significance for underrepresentation in *CFTR*^*−/−*^ animals.

We then considered the independent contribution of the two distal lung samples from the cloned *CFTR*^*−/−*^ lambs (AXH144, AXH145) which might alter the proportions compared to the four non-cloned ones. The Seurat analysis was repeated without these two cloned samples and the proportions test again showed overrepresentation of ciliated cells (among others) in *CFTR*^*−/−*^ animals. Similarly, T cells and neutrophils were underrepresented in the non-cloned *CFTR*^*−/−*^ animals alone and a third immune cell cluster, possibly alveolar macrophages reached statistical significance (data not shown).

### Minor overrepresentation of immune cells in proximal lung of *CFTR*^*−/− *^sheep at 120 days of gestation

**Fig. 3:**
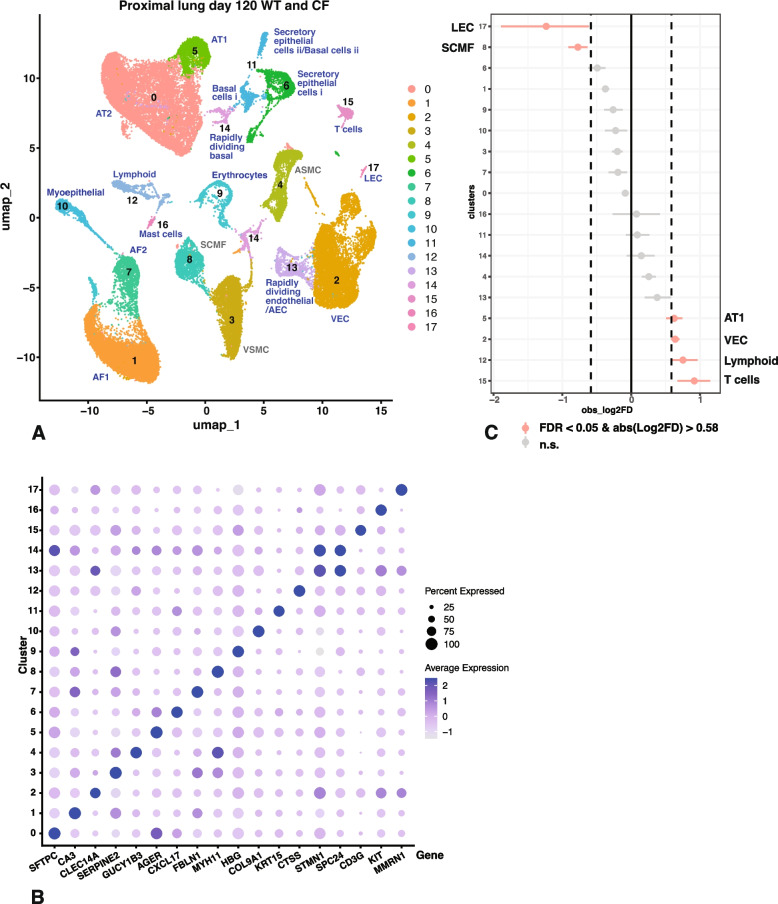
Single cell RNA-seq defines cell types in 120 day WT and *CFTR*^*−/−*^ sheep proximal lung and shows slight overrepresentation of immune cells in *CFTR*^*−/−*^ tissues. **A** UMAP plot of merged data from 4 WT and 7 *CFTR*^*−/−*^ donors identifies 18 clusters by differential gene expression profiles, each named by cell type. Abbreviations are defined in the legends to Figs. 1 and 2. The mesenchymal clusters in grey denote ambiguous identity. **B** The dotplot shows expression of one marker for each cell type/cluster identified in **A** and described in the text: 0, *SFTPC*; 1, *CA3*; 2, *CLEC14A*; 3, *SERPINE2*; 4, *GUCY1B3*; 5, *AGER*; 6, *CXCL17*; 7, *FBLN1*; 8, *MYH11*; 9, *HBG*; 10, *COL9A1*; 11, *KRT15*; 12, *CTSS*; 13, *STMN1*; 14, *SPC24*; 15, *CD3G*; 16, *KIT*; 17, *MMRN1*. **C** Single cell proportions test comparing *CFTR*^*−/−*^ to WT shows significant overrepresentation of T cells (15) and other lymphoid cells (12) and slight but statistically significant underrepresentation of SCMF cells (8) in *CFTR*^*−/−*^ tissues

Next, we investigated the cellularity of WT and *CFTR*^*−/−*^ sheep proximal lung tissue at 120 days of gestation, equivalent to > 30 weeks of human gestation and during the mid-saccular stage of lung development in both species. We used a resolution of 0.1 in the Seurat pipeline with the filtering parameters stated in the methods section and identified 18 cell clusters in the merged WT and *CFTR*^*−/−*^ proximal lung samples at 120 days. Cluster resolution by identity is shown in Fig. 3A and by sample in Fig. S2C. Clusters were assigned a cellular identity by inspection of the marker gene lists shown in Table S4 and correlation with known gene expression patterns in the human proximal lung, and are shown in the dot plot in Fig. 3B. Due to the incomplete differentiation of the lung at 120 days of gestation and the partial annotation of the sheep genome the unequivocal identification of all clusters was challenging. However, we again performed additional validation of cluster assignment using LungMAP (Guo et al. 2023) and post-hoc compared our data with the human fetal lung atlas resource, which contains data up to about 22 weeks of gestation (He et al. 2022).

i) *Cluster identity by differential gene expression profiles in proximal lung at 120 days of gestation.*

The most abundant cluster 0 are AT2 cells, with surfactant genes *SFTPC*, *SFTPD* and *SFTPB* and *SLC34A2*. We previously observed high levels of surfactant gene expression by 120 days in bulk RNA-seq data from sheep fetal lung (Kerschner et al. 2023) though the earlier data did not identify the cellular source of transcript. Although cluster 0 cells have the same marker genes as AT2 cells it is also possible that they are in fact a developmental precursor cell type. Close to cluster 0 in the UMAP space is cluster 5, which likely encompasses AT1 cells, as they have the classical markers *AGER*, *RTKN2*, *CLD18*, *EMP2* and *KRT7*, and are negative for *ATP6V1C2* and *ACSL4*. Clusters 1, 3, 4, 7 and 8 are all mesenchymal cell types observed in the sheep fetal lung consistent with their critical role in lung development (reviewed in (El Agha and Thannickal 2023)). They are somewhat difficult to correlate with specific cell types in the term lung due to plasticity in gene expression profiles during development. Cluster 1 cells have markers of AF1 cells that include *CA3*, mesenchyme homeobox 2 (*MEOX2*), *GPC3*, *ITGA8*, olfactomedin like 3 (*OLFML3*), *TCF21* and *COL13A1*, but also vascular cell adhesion molecule 1 (*VCAM1*), though they lack *MYH11* and *TGFBI*. Cluster 7 cells, which are close to cluster 1 in UMAP space are likely AF2 cells, with marker genes *COL1A1*, *COL1A2*, *MFAP5*, *PCOLCE*, *PI16*, *TCF2*1, *DCN* and *GPC3*. We were unable to assign a definitive identity to clusters 3, 4 and 8 mesenchymal cells and so highlighted them in grey font in Fig. 3A. Cluster 4 cells are likely ASMC, with marker genes including caldesmon 1 (*CALD1)*, *ACTA2*, *DES*, *MYH11* and guanylate cyclase 1 soluble subunit beta 3 (*GUCY1B3*), though *LGR6* is not clearly annotated in the sheep genome. Cluster 3 has marker genes consistent with VSMC, while high expression of *FGF18* in cluster 8 is indicative of SCMF. Cluster 10 may be myoepithelial cells (MEC) marked by *COL9A1*, *COL9A2*, *COL9A3*, and *COL11A1* or immature chondrocytes as hyaluronan and proteoglycan link protein 1 (*HAPLN1*) and biglycan (*BGN*) are also on the marker gene list. Cluster 2 and 13 are both endothelial cell types with marker genes for cluster 2 suggesting VEC and including *RAMP2*, *PECAM1*, *CDH5*, endothelial cell surface expressed chemotaxis and apoptosis regulator (*ECSCR*), *CD34* and 15-hydroxyprostaglandin dehydrogenase (*HPGD*), which is also particularly highly expressed in capillary type 2 (CAP2) cells. The identity of cluster 13 is uncertain as it shares AEC markers with those of rapidly dividing cells, such as *STMN1* (encoding stathmin). Moving forward to cells with a clearer identity, cluster 6 cells are likely secretory epithelial cells with *TFF3*, vitelline membrane outer layer 1 homolog (*VMO1*), *EHF*, *CLDN10* and *CXCL17* on the marker gene list. Cluster 11 have markers of basal cells such as *S100A2* and *EHF*, though also of goblet cells notably anterior gradient 2, protein disulphide isomerase family member (*AGR2*) and TFF3. The cluster is in 2 regions of UMAP space indicating cellular heterogeneity, which may correlate with different basal cell states. Notably the basal stem cell marker TP63 is not on the marker gene list for cluster 11, though it is expressed in a subset of cells in the cluster named "Basal cells i" in Fig. 3A. Cluster 12 cells are lymphoid (non T-cell) expressing high levels of *CD68*, *FCER1G*, transmembrane immune signaling adaptor TYROBP (*TYROBP*), *CSFIR,* interleukin 2 receptor subunit gamma (*IL2RG*) and *CD53* but lacking *PTPRCAP*. Cluster 14 includes rapidly dividing cells of uncertain identity. Cluster 15 are T cells, with *CD3E* and *CD3G* high on the marker gene list. Cluster 16 are mast cells with *TPSB2*, *KIT, FCER1A* and *S100A10* on the marker gene list, while cluster 17 are LEC cells as evidenced by *PROX1* and *MMRN1* markers.

ii) *Cluster abundance in proximal lung of WT and CFTR* ^*-/-*^ *sheep at 120 days of gestation.*

When we compared the relative abundance of individual clusters in 120 day proximal lung from *CFTR*^*−/−*^ and WT animals (Fig. 3C, again FDR < 0.05 and absolute log2 FD > 0.58) the differences were less apparent than at term. Statistically overrepresented clusters in *CFTR*^*−/−*^ tissues included T cells (cluster 15) and other lymphoid cells (cluster 12) with VEC in cluster 2 and AT1 cells in cluster 5 just reaching statistical significance. The mesenchymal cells in cluster 8 (likely SCMF) reach statistical significance for underrepresentation together with LECs, though the large error bar in the latter renders it of doubtful significance. Of note, when we repeated the Seurat analysis to exclude the two non-cloned 120 day *CFTR*^*−/−*^ animals (AXH134/135) the results of the proportions test were similar to the overall analysis, with overrepresentation of T cells and other lymphoid cells and underrepresentation of a mesenchymal cell cluster, likely SCMF.

### Overrepresentation of immune cells in distal lung of* CFTR*^*−/−*^ sheep at 120 days of gestation

**Fig. 4:**
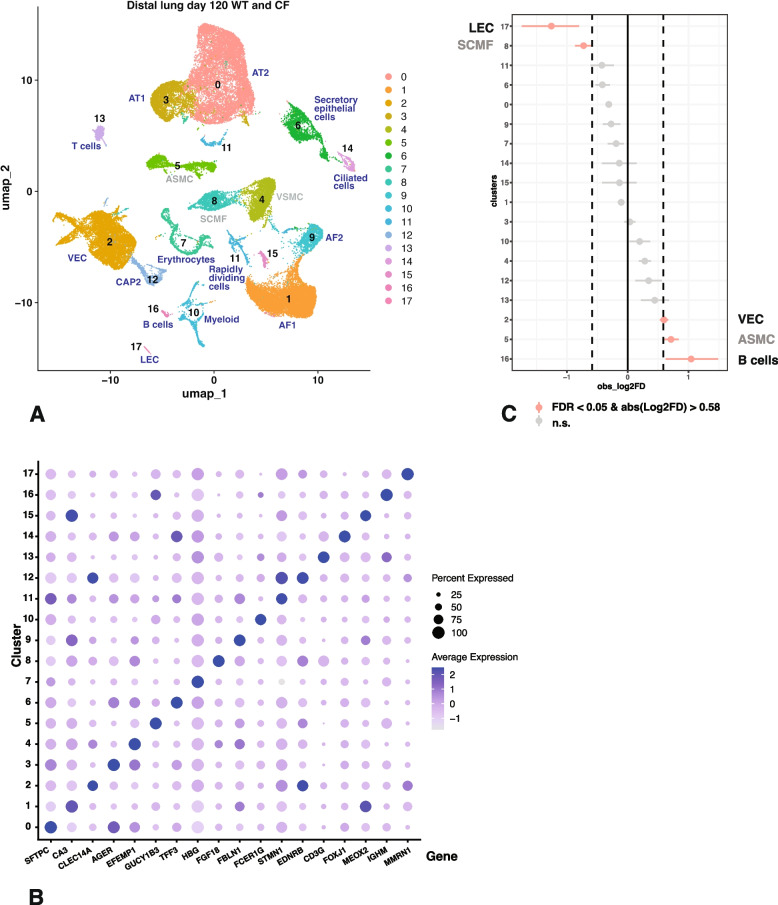
Single cell RNA-seq defines cell types in 120 day WT and *CFTR*^*−/−*^ sheep distal lung and shows slight overrepresentation of B cells in *CFTR*^*−/−*^ tissues. **A** UMAP plot of merged data from 4 WT and 7 *CFTR*^*−/−*^ donors identifies 18 clusters by differential gene expression profiles, each named by cell type. Abbreviations are defined in the legends to Figs. 1 and 2 except CAP2, capillary type 2 cells. The mesenchymal clusters in grey denote ambiguous identity. **B** The dotplot shows expression of one marker for each cell type/cluster identified in **A** and described in the text: 0, *SFTPC*; 1, *CA3*; 2, *CLEC14A*; 3, *AGER*; 4, *EFEMP1*; 5, *GUCY1B3*; 6, *TFF3*; 7, *HBG*; 8, *FGF18*; 9, *FBLN1*; 10, *FCER1G*; 11, *STMN1*; 12, *EDNRB*; 13, *CD3G*; 14, *FOXJ1*; 15, *MEOX2*; 16, *IGHM*; 17, *MMRN1*. **C** Single cell proportions test comparing *CFTR*^*−/−*^ to WT shows slight but statistically significant overrepresentation of B cells (16) and ASMC (5) and slight underrepresentation of SCMF cells (8) in *CFTR*^*−/−*^ tissues

The cellularity of WT and *CFTR*^*−/−*^ sheep distal lung tissue at 120 days of gestation was investigated with a resolution of 0.1 in the Seurat pipeline and using the filtering parameters described in the methods. UMAP plots show cluster resolution by identity in Fig. 4A and by sample in Fig. S2D. Eighteen clusters were assigned a cellular identity by inspection of the marker gene lists shown in Table S5 and correlation with known gene expression patterns in the human lung. Cluster marker genes are shown by dot plot in Fig. 4B. As before, cluster validation relied upon LungMAP (Guo et al. 2023) and the fetal human fetal lung atlas resource (He et al. 2022).

i) *Cluster identity by differential gene expression profiles in distal lung at 120 days of gestation.*

Consistent with our data on the proximal lung at 120 days the most abundant cluster 0 included AT2 cells, with multiple surfactant genes at the top of the marker list including *SFTPC*, *SFTPD*, *SFTPB*, *SFTPA1* and *SFTA*, together with *SLC34A2*, though lacking *ALSL4* and *ATP6V1C2*. Adjacent in UMAP space is cluster 3, which includes AT1 cells as shown by *AGER*, *RTKN2*, *EMP2* and *CLDN18* on the marker gene list. Cluster 1 are AF1 cells, with *TCF21*, *MEOX2*, *VCAM1*, *COL13A1* and *ITGA8* on the marker gene list but absence of *MYH11* and *TGFBI*. Cluster 15 has similar marker genes. Close to cluster 1 in UMAP space is cluster 9, with markers of AF2 cells including *DCN*, *COL1A1*, *COL1A2*, *MFAP5*, *COL14A1*, *GPC3* and *MGP*. Cluster 2 are likely VEC, with *CLEC3B*, *PECAM1*, *CDH5* and *CD34* all high on the marker gene list. The adjacent cluster 12 has markers of CAP2 cells, specifically endothelin receptor type B (*EDNRB*), *HPGD* and *RAMP2* though some markers seen in other cell types are also evident, including *STMN1* and several tubulins (*TUBA1B* and *TUBB4B*). As in the proximal lung at 120 days, the multiple mesenchymal cell clusters including clusters 4 (in particular), 5 and 8 were difficult to assign an unequivocal identity, and so are labelled in grey font in Fig. 4A. Cluster 4 cells have some similar markers to SCMF cells, although *FGF18* is expressed in cluster 8 not cluster 4 cells suggesting the latter are VSMC. Cluster 5 cells are likely ASMCs with *DES*, *ACTA2*, *MYH11*, *TPM*, *TAGLN* and *MYLK* on the marker gene list. Cluster 8 are probably SCMF with *CNN1*, *ACTA2* and *MYL6* as markers. Cluster 6 cells are secretory epithelial cells, marked by *TFF3*, *SCGB2A2*, *EHF*, *KLF5*, *SCGB3A2* and *SOX2*, and lacking EGF like domain multiple 6 (*EGFL6*) and *ETV5*. Cluster 7 are immature red blood cells with *HBG*, *HBA1* and *HBM* at the top of the marker gene list. Clusters 10, 13 and 16 are all immune cell types. Cluster 10 cells are likely myeloid, possibly alveolar macrophages expressing high levels of *FCER1G*, *TYROBP* and *CD53* but lacking *PTPRCAP,* although neither *CD68* nor *CSF1R* are markers. Cluster 13 are T cells with *CD3E*, *CD3G*, *PTPRCAP* and *PTPRC* on the marker gene list. Cluster 16 have markers that are consistent with B lymphocytes, with *CD79A* and *CD79B* and multiple HLA- genes including -*DQA*, *DQB* and *DMB* on the list, though the presence of certain other markers suggests other antigen presenting cells may be included in the cluster or they are immature. Cluster 11 cells have similarities with rapidly dividing cells that we observed in sheep fetal pancreas and are also present in other lung scRNA-seq data (Paranjapye et al. 2022a). These cells have marker genes associatesd with cell proliferation and cell division such as topoisomerase II alpha (*TOP2A*), TPX2 microtubule nucleation factor (*TPX2*) and multiple centromeric components (*CENPF*, *CENPW* and *CENPE*). Cluster 14 cells are FOXJ1-expresssing multiciliated cells. Cluster 17 are LECs marked by *PROX1*, *MMRN1*, CC motif chemokine ligand 2 (*CCL2*), neuropilin 1 (*NRP1*) and *PTX3*, but lacking *CLEC14A*, *SOX17*, *EMCN* and *CLEC1A*.

ii) *Cluster abundance in distal lung of WT and CFTR*^*-/-*^*sheep at 120 days of gestation.*

Lastly, we compared the relative abundance of individual clusters in 120-day distal lung from *CFTR*^*−/−*^ and WT animals (Fig. 4C, again FDR < 0.05 and absolute log2 FD > 0.58) and as for the proximal lung the differences were less apparent than at term. Statistically overrepresented clusters in the *CFTR*^*−/−*^ animals included the presumptive B cells in cluster 16, though the error bar on these cells was large and ASMC (cluster 5) and VEC (cluster 2) just reached statistical significance. Clusters that were statistically underrepresented in *CFTR*^*−/−*^ animals were LECs (cluster 17), though again the error bar on this small cluster was large, and SCMF in cluster 8. We also reanalyzed the data for d120 distal lung without the two non-cloned *CFTR*^*−/−*^ animal samples (AXH132,133) and compared the relative abundance of individual clusters in *CFTR*^*−/−*^ and WT animals. Consistent with the complete data set, B cells were the most overrepresented in *CFTR*^*−/−*^ animals, though the error bar was large, together with ASMC and T cells also just reaching statistical significance. The only significantly underrepresented cluster in this comparison was LECs, though the error bar was very large (data not shown).

### Cellular imbalance at 80 days gestation in proximal and distal lung of* CFTR*^*−/− *^sheep

The cellularity of WT and *CFTR*^*−/−*^ sheep proximal and distal lung tissue at 80 days of gestation was investigated with a resolution of 0.2 in the Seurat pipeline and using the same filtering parameters as for later timepoints. Cluster assignment was more challenging than at 120 days gestation and term due to the immaturity of many cell types.

i) *Cluster identity by differential gene expression profiles in proximal lung at 80 days of gestation.*

**Fig. 5:**
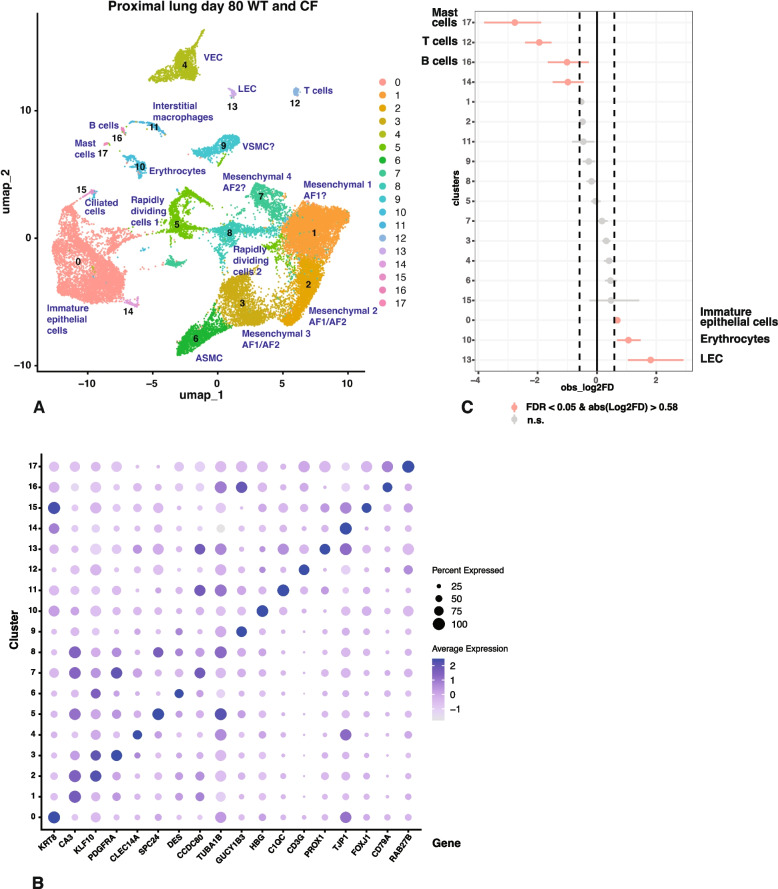
Single cell RNA-seq defines cell types in 80 day WT and *CFTR*^*−/−*^ sheep proximal lung. **A** UMAP plot of merged data from 2 WT, 2 *CFTR*^+/−^ and 3 *CFTR*^*−/−*^ donors identifies 18 clusters by differential gene expression profiles, each named by cell type. Abbreviations are defined in the legend to Fig. 1. The *CFTR*^+/−^ animals were grouped with WT as they have WT phenotypes. Multiple mesenchymal clusters have uncertain identity due incomplete differentiation. **B** The dotplot shows expression of one marker for each cell type/cluster identified in **A** and described in the text: 0, *KRT8*; 1, *CA3*; 2, *KLF10*; 3, *PDGFRA* 4, *CLEC14A*; 5, *SPC24*; 6, *DES*; 7, *CCDC80*; 8, *TUBA1A*; 9, *GUCY1B3*; 10, *HBG*; 11, *C1QC*; 12, *CD3G*; 13, *PROX1*; 14, *TJP1*; 15, *FOXJ1*; 16, *CD79A*; 17, *RAB27B*. **C** Single cell proportions test comparing *CFTR*^*−/−*^ to WT shows underrepresentation of mast cells, T and B cells in *CFTR*^*−/−*^ animals. The larger error bar on the overrepresented LEC suggests large sample variation

We identified 18 cell clusters in proximal lung at 80 days (Fig. 5A) and sample contributions to each cluster are shown in Fig. S5A. The marker gene list for each cluster is shown in Table S6, and a dot plot summarizing key marker genes for each cluster is shown in Fig. 5B. The most abundant cluster 0 includes a mixture of immature epithelial cells identified by *KRT8*, *KRT19*, *KRT18* and *CLDN*4, but markers of AT1 (*AGER*), AT2 (*SFTPC*) and secretory epithelial cells (*KLF5*) are also evident. Close to cluster 0 in the UMAP space are clusters 14 and 15. Cluster 15 are ciliated cells, marked by *FOXJ1* and *RSPH1*, but cluster 14 was not identified and contained many mitochondrial markers that passed the filter cut off. Clusters 1, 2, 3 and 7 are all mesenchymal cell types that share many markers in common thus making it difficult to definitively assign a fully differentiated cell identity to each of them. It is likely that they represent immature cells that have multipotency at 80 days gestation. Cluster 1 has many markers of AF1 cells, including *CA3*, *TCF21*, protein phosphatase 1 regulatory inhibitor subunit 14A (*PPP1R14A*), which regulates myosin phosphorylation and hence smooth muscle contraction, *GPC3* and *PCOLCE2*. Cluster 2 has only 21 marker genes reaching the 0.7 cutoff for myAUC value and only the lncRNA *GTL2*/*MEG3* is not shared with the longer cluster 1 list, again suggesting that cluster 2 cells may be an immature cell type. Cluster 3 cells also have very few marker genes and these intersect with AF1 cells, AF2 cells (*ROBO2*) but are most similar to SCMF cells with *PDGFRA*, *TGFBI* and *SDC2* on the list. Since SCMF cells are a transient population associated with alveogenesis this suggests the process may have already commenced at 80 days gestation or that this cell population has additional functions earlier in gestation. Markers of cluster 7 cells are consistent with AF2 cells and include *DCN*, *MFAP5*, *CCDC80* and *PCOLCE*. Cluster 4 cells are VEC, identified by *CLEC14A*, *CDH5*, *SOX17*, *PECAM1* and *EMCN*. Many markers of the cell cycle, cell division and DNA replication identify both clusters 5 and 8 as distinct populations of rapidly dividing cells with no specific lung function, for example SPC24 component of NDC kinetochore complex (*SPC24*). Cluster 6 appears to be ASMC consistent with *ACTA2*, *MYLK*, *MYH11, TAGLN*, *TPM2* and *DES* among marker genes, while cluster 9 cells have many of the same markers but also *GUCY1B3*, potassium inwardly rectifying channel subfamily J member 8 (*KCNJ8*) and *NOTCH3* consistent with either VSMC or pericytes. Cluster 10 are contaminating immature erythrocytes as shown by multiple hemoglobin subunit genes on the marker list, and cluster 11 are interstitial macrophages identified by *CIQC, C1QB* and *CD163*. T cells comprise cluster 12, with *CD3E*, *CD3G*, *PTPRC* and *PTPRCAP* among marker genes and cluster 13 are LECs, characterized by *PROX1* and *MMRN1*. Lastly, cluster 16 are B cells, with *CD79A*, *CD79B*, *CD37* and multiple HLA components among marker genes, and cluster 17 are likely mast cells identified by *TPSB2*, *FCER1G, LTC4S* and *KIT*.

ii) *Cluster abundance in proximal lung of WT and CFTR* ^*-/-*^ *sheep at 80 days of gestation.*

Next considering the single cell proportions test comparing day 80 proximal lung cells from WT and *CFTR*^*−/−*^ animals (Fig. 5C), we note that error bars are generally greater on all clusters than on the equivalent comparisons at later gestational time points. Hence, we are not confident in their biological significance, despite statistical significance. LEC cells are overrepresented in *CFTR*^*−/−*^ animals and immature epithelial cells are just statistically more abundant. The overrepresentation of erythrocytes is simply a reflection of the varying efficacy of the red blood cells depletion during the single cell preparation protocol. Apparently significantly underrepresented in *CFTR*^*−/−*^ animals are mast cells, T cells and B cells.

iii) *Cluster identity by differential gene expression profiles in distal lung at 80 days of gestation.*

**Fig. 6:**
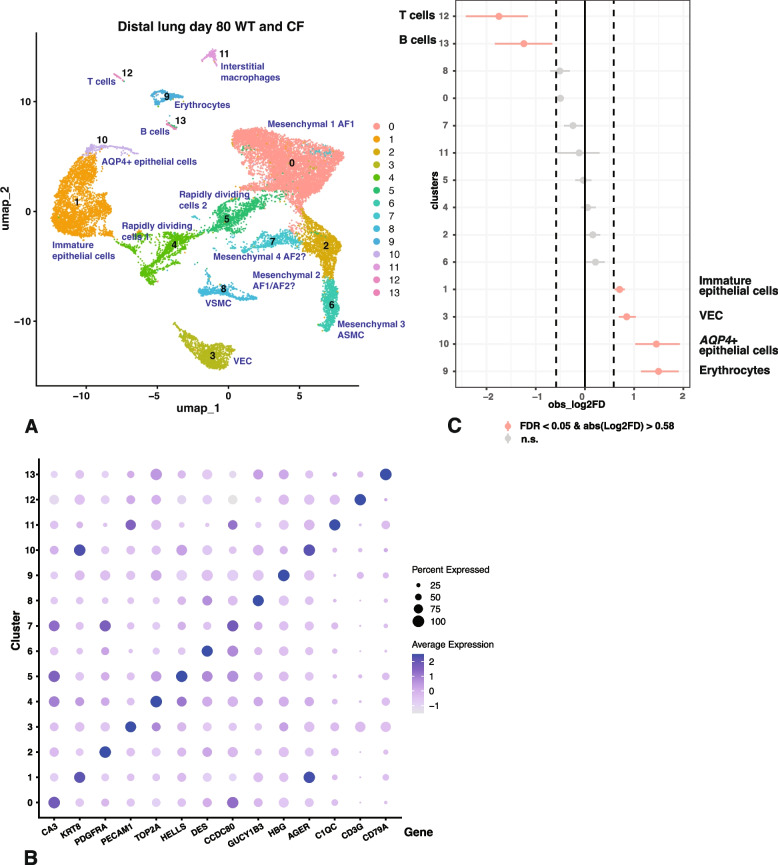
Single cell RNA-seq defines cell types in 80 day WT and *CFTR*^*−/−*^ sheep distal lung. **A** UMAP plot of merged data from 2 WT, 2 *CFTR*^+/−^ and 3 *CFTR*^*−/−*^ donors identifies 14 clusters by differential gene expression profiles, each named by cell type. Abbreviations are defined in the legend to Fig. 1. Multiple mesenchymal clusters have uncertain identity due incomplete differentiation. **B** The dotplot shows expression of one marker for each cell type/cluster identified in **A** and described in the text: 0, *CA3*; 1, *KRT8*; 2, *PDGFRA*; 3, *PRCAM1*; 4, *TOP2A*; 5, *HELLS*; 6, *DES*; 7, *CCDC80*; 8, *GUCY1B3*; 9, *HBG*; 10, *AGER*; 11, *C1QC*; 12, *CD3G*; 13, *CD79A*. **C** Single cell proportions test comparing *CFTR*^*−/−*^ to WT shows statistical overrepresentation of *AQP4* + epithelial cells, VEC and immature epithelial cells and underrepresentation of T and B cells, though the large error bars suggest substantial variation between samples

Lastly, we examined distal lung tissue at 80 days gestation and only identified 14 clusters at a resolution of 0.2 (Fig. 6A) with the contribution by donor to each cluster shown in Fig. S5B. The marker gene list for each cluster is shown in Table S7, and a dot plot summarizing key marker genes for each cluster is shown in Fig. 6B. The largest cluster 0 are AF1 cells with markers *CA3*, *PPP1R14A*, *TCF21*, *COL13A1* and *GPC3*. Clusters 2, 6, 7 and 8 are also mesenchymal cells. Cluster 6 are ASMC with markers including *ACTA2*, *DES*, *TPM2*, *TAGLN* and *MYH11*, cluster 7 are likely AF2 cells with *CCDC80*, *DCN* and multiple collagen genes including *COL1A1* and *COL1A2* on the marker gene list, cluster 8 are probably VSMCs sharing many markers with ASMCs but also marked by *GUCY1B3*, *KCNJ8* and *NOTCH3*. Cluster 2 cells have a short marker gene list and are more difficult to identify, they share markers of AF1 and AF2 cells but also have *PDGFRA*, *TGFBI* and *SDC2* as markers consistent with SCMF cells. Cluster 1 are immature epithelial cells with markers of secretory epithelial cells, AT1 and AT2 cells, and cluster 10 which is nearby in UMAP space are also immature epithelial cell that are marked by *AQP4* consistent with either basal or ciliated cells. Cluster 3 are VEC marked by *CDH5* and *PECAM1,* though some markers of fully differentiated VEC such as *SOX17* and *CLEC14A* are not on the marker list. Two distinct population of rapidly dividing cells, marked by genes involved in the cell cycle, cell division and DNA replication comprise clusters 4 and 5, which are similar to clusters 5 and 8 in the proximal lung at 80 days gestation and together may indicate a period of rapid tissue growth and differentiation. Cluster 9 are erythrocytes, marked by many hemoglobin subunit genes and 11 are interstitial macrophages, marked by *C1QC*, *C1QA*, *C1QB* and CD163. Cluster 12 are T cells, with *CD3E* and *CD3G* as characteristic markers and cluster 13 are B cells marked by *CD79A* and several HLA component genes.

iv) *Cluster abundance in distal lung of WT and CFTR* ^*-/-*^ *sheep at 80 days of gestation.*

When comparing the day 80 distal lung from WT and *CFTR*^*−/−*^ animals in a single cell proportions test (Fig. 6C), as for the proximal lung at this gestational age the error bars are large and so the results must be interpreted with caution. Immature epithelial cells, VEC and AQP4 + epithelial cells reach statistical significance for overrepresentation in *CFTR*^*−/−*^ animals as do erythrocytes, though the latter are an artifact of less efficient removal of these cells in the protocol. Underrepresented cell clusters in *CFTR*^*−/−*^ animals include B cells and T cells.

### Immunocytochemical validation of ciliated and basal cell imbalance in* CFTR*^*−/−*^animals at term

**Fig. 7:**
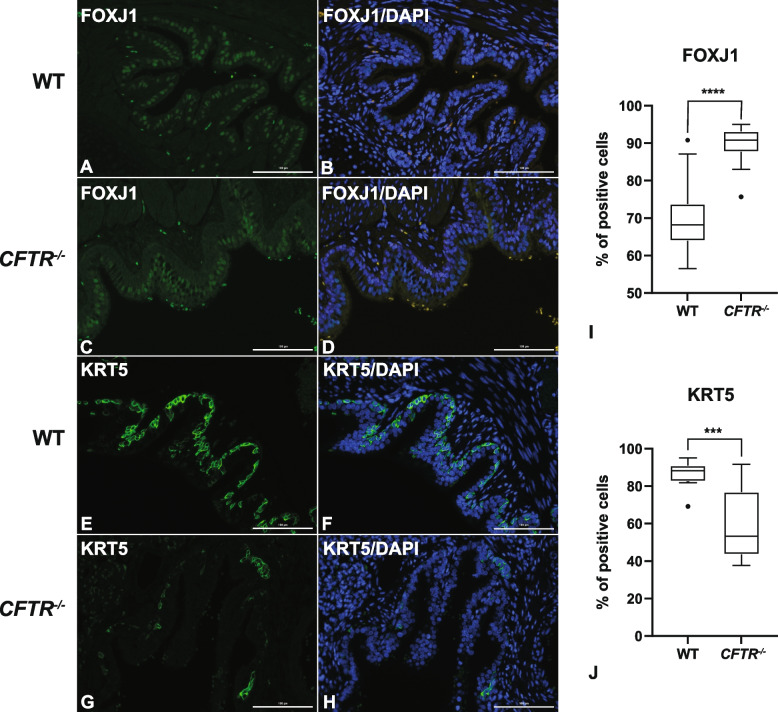
Immunochemistry shows overrepresentation of FOXJ1+ ciliated cells and underrepresentation of KRT5+ basal cells in *CFTR*^*−/−*^ bronchiolar epithelia compared to WT at term in proximal lung. Representative images show FOXJ1 (**A**-**D**) and KRT5 (**E**–**H**) expression in the bronchioles of proximal lung sections from WT (**A**, **B**, **E**, **G**) and *CFTR*^*−/−*^ (**C**, **D**, **F**, **H**) animals. (**A**, **C**) FOXJ1 reactivity is shown in green; (**B**, **D**) overlay images display FOXJ1 signal and DAPI nuclear counterstain in blue. Autofluorescent signals from red blood cells and some immune cells appear as yellow/orange. (**E**, **G**) KRT5 reactivity is shown in green; (**F**, **H**) overlay images display KRT5 signal and DAPI nuclear counterstain in blue. Scale bar in all panels = 100 μm. Quantification of FOXJ1 (**I**) and KRT5 (**J**) + cells as a percentage of all apical or basolateral cells, respectively (for full details see methods) from proximal lung sections of 3 WT and 3 *CFTR*^*−/−*^ animals. Statistics: unpaired *t*-test **** *p* < 0.0001; *** *p* = 0.0002

To establish whether the overrepresentation of ciliated cells and underrepresentation of basal and secretory epithelial cells identified in the *CFTR*^*−/−*^ proximal lung at term by scRNA-seq could also been seen by immunochemistry, we used antibodies specific to FOXJ1, KRT5 and SCGB3A2, respectively (Fig. 7, Fig. S6). For quantitation we used the Lionheart FX automated microscope and Gen5 software as described fully in the methods. Cells that expressed a specific marker (in green, Fig. 7A, C, E, G) and all DAPI-stained nuclei (in blue, Fig. 7B, D, F, H) in the surface epithelial layer of small airways (bronchioles) were counted in each field. The percentages of FOXJ1-positive ciliated epithelial cells and KRT5-positive basal cells were calculated by dividing the number of FOXJ1- or KRT5-positive cells by the total number of DAPI-positive apical or basal cells, respectively. Lung sections from three WT and three *CFTR*^*−/−*^ animals at term were analyzed and five or more small airways were randomly selected from each slide. Of note, ciliated cells were seen in all airways with diameters of > ~ 100 µm, though basal cells were absent from airways < ~ 250 µm. Approximately equal numbers of cells were counted in WT and *CFTR*^*−/−*^ samples. The data in Fig. 7I and J include a total of more than 1100 cells assayed for FOXJ1 expression and more than 800 cells assayed for KRT5 expression. The graphs show statistical overrepresentation of ciliated cells (*p* < 0.0001) and underrepresentation of basal cells (*p* = 0.0002) when comparing *CFTR*^*−/−*^ with WT samples, consistent with the scRNA-seq data. Of note, this difference was only seen in small airways (100 µm-2 mm) and not in larger, cartilaginous airways in which ciliated and basal cells were equally abundant in the two genotypes. The distribution of SCGB3A2 expressing secretory cells in the surface epithelium of bronchioles (Fig. S6) was difficult to quantify due to the more diffuse cytoplasmic staining pattern, in comparison to the clear nuclear stain of FOXJ1 and cytoskeletal stain of KRT5. However, the overall number of SCGB3A2 expressing cells appeared consistently lower in the epithelial layer lining bronchioles of *CFTR*^*−/−*^ animals than WT, concurring with the scRNA-seq data.

### Pseudotime analysis

**Fig. 8:**
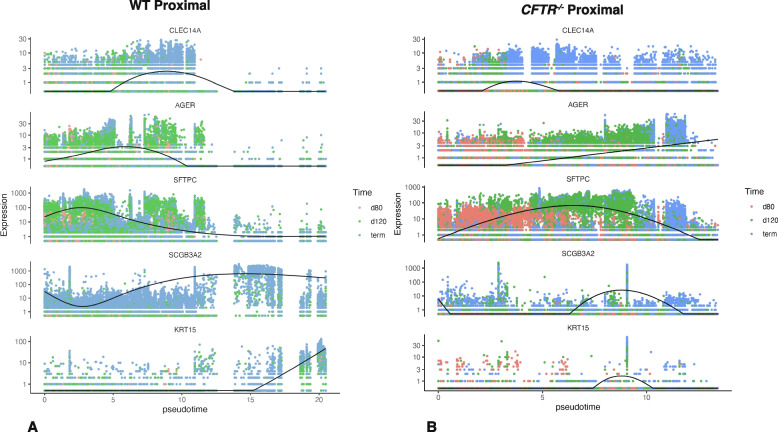
Pseudotime analysis of scRNA-seq data using Monocle 3 shows altered gene expression profiles in WT and *CFTR*^*−/−*^ sheep proximal lung. Markers for AT1 cells (*AGER*), AT2 cells (*SFTPC*), secretory epithelial cells (*SCGB3A2*) and basal cells (*KRT15*) show different splines in WT and *CFTR*^*−/−*^ tissues, while a marker for VEC (*CLEC14A*) has similar profiles in the two genotypes

We next use Monocle 3 to perform pseudotime analysis on the developmental trajectories of WT and *CFTR*^*−/−*^ proximal and distal sheep lung tissue to compare differences between the two genotypes. Albeit we have real-time gene expression data at specific time points through gestation it was of interest to monitor the progress of individual cell types through pseudotime. This analysis uses the scRNA-seq data in an unbiased way to show the dynamics of transcriptome development in different cells, hence providing cell type trajectory information across the complete developmental time course after 80 days gestation. To achieve this, we chose a marker gene for each cluster and plotted expression against pseudotime (Fig. 8, Figs. S7 and S8). In proximal lung the spline (representing mean expression as a function of pseudotime) for the VEC marker *CLEC14A* is rather similar in WT and *CFTR*^*−/−*^ animals (Fig. 8). In contrast, the splines markers for AT1 cells (*AGER*), AT2 cells (*SFTPC*), secretory epithelial cells (*SCGB3A2*) and basal cells (*KRT15*) show very different profiles. *AGER*- and *SFTPC*-expressing cells are more abundant earlier in pseudotime in WT animals than in *CFTR*^*−/−*^ animals, although it is possible that *AGER* is also marking immature alveolar cells or AT0 cells in addition to AT1 cells. The splines for secretory epithelial cells and basal cells show an increase through pseudotime in WT animals while *CFTR*^*−/−*^ animals show a transient spline, consistent with our observation that these two cell types are significantly underrepresented in *CFTR*^*−/−*^ animals at term (Fig. 1B). In distal lung VEC cells show a slightly different profile in WT and *CFTR*^*−/−*^ animals (Fig. S7) while the splines for AT1, AT2, secretory epithelial, and basal cells are rather similar in the two genotypes. These observations reflect the single cell proportions test in Fig. 2B. However, markers for SCMF (*SERPINE2*), AF1 cells (*CA3*), ASMC (*ACTA2*) and VSMC (*TPM1*) show splines with very different, almost reciprocal profiles in the WT and *CFTR*^*−/−*^ animals (Fig. S8). The significance of these alterations in profiles of multiple stromal cell types in pseudotime is unclear. Markers for less abundant cell types such as ciliated cells and immune cells (lymphocyte sub-types) did not generate meaningful splines due to the low expression levels.

## Discussion

The CF sheep models were generated with the specific goal of examining early developmental events, which cannot be examined rigorously in human but could significantly impact lung health at birth and postnatally (Harris 1997; Fan et al. 2018; Viotti Perisse et al. 2021; Van Wettere et al. 2022). In this context we must accept the incomplete annotation of the sheep genome which slightly limits the power of scRNA-seq data analysis. We used human scRNA-seq resources to aid in defining cell identity (Regev et al. 2017; Hu 2019; Sun et al. 2022; Guo et al. 2023; Sikkema et al. 2023) and also performed comparative analyses with the time course of early human gestation (He et al. 2022), which will be presented elsewhere. The availability of these robust resources generates further support for the interpretation of the results on the sheep lung that we present here.

Since there are no prior scRNA-seq datasets available on sheep lung we first generated cell atlases for proximal lung and distal lung at each developmental time point, combining WT and *CFTR*^*−/−*^ animals. We present UMAP plots at resolutions (0.2 for term and 80 days and 0.1 for 120 days) that enabled the identification of the maximum number of cell clusters, based upon the marker gene lists, though additional resolutions were also used to investigate the contribution of other cell types. Unequivocal cluster assignment was achieved only in term tissue as there was some ambiguity in cell clusters in the 120 day and 80 day samples. In both regions of the term lung the most abundant cells were VEC, followed by AT1, SCMF and AT2 cells in the proximal lung, and SCMF, AT1 and AT2 cells in the distal lung. In contrast in the 120 day proximal and distal lung AT2 cells were the most abundant, followed by AF1, VEC and VSMC in the proximal lung and AF1, VEC and AT1 cells in the distal lung. At 80 days, immature epithelial cells comprised the most abundant cluster in proximal lung followed by several mesenchymal cell types, while in the distal lung mesenchymal cells were the largest cluster, followed by immature epithelial cells, other mesenchymal cells with an ambiguous identity and VECs. Together these data reflect the changes in cellularity of the developing sheep lung.

The most challenging cluster identification was that of stromal cells/mesenchymal cells in the developing lung, in part due the incomplete annotation of the ovine genome resulting in a lack of negative markers to exclude certain clusters. Similarly, detailed identification of different immune cell clusters was sometime difficult, so in those cases we present the main cell types without detailed subclustering. Apart from these equivocal cluster assignments, we generate a lung cell atlas that has many similarities with the human lung and thus supports that utility of the sheep model to study aspects of human lung development and disease.

As our goal was to investigate CF disease-associated changes in the *CFTR*^*−/−*^ lung through development and at birth, we next used the cell atlas data in proximal and distal lung at each time point to compare WT and *CFTR*^*−/−*^ tissues. There are multiple ways to perform such a comparison, though here we use the simplest metric of comparing the relative contribution of WT and *CFTR*^*−/−*^ cells to each defined cluster at each timepoint, by performing single cell proportions tests (Miller et al. 2021). Although we interpret the single cell proportions tests with caution, given the possibility of sampling bias, the large number of cells and of tissue samples that we analyzed at each time point give confidence to our conclusions. The results suggest the most significant differences between the *CFTR*^*−/−*^ and WT lung tissues are seen in the proximal regions of the lung (note this defines a lung region and is not a reference to airway diameter).

First considering epithelial cell populations, at 80 days of gestation immature epithelial cells just reach statistical significance for overrepresentation in both regions of the lung, as do the small population of *AQP4-*expressing epithelial cells only in distal lung. At 120 days the only epithelial cell population passing the cut off for statistical significance are AT1 cells which are slightly overrepresented only in the *CFTR*^*−/−*^ proximal lung. Perhaps the most biologically significant changes in epithelial cell populations are seen at term, when FOXJ1-expressing ciliated cells are the most overrepresented cell type in both proximal and distal lung regions of *CFTR*^*−/−*^ lambs. AT1 cells in the term proximal lung and cells with markers of both AT1 and AT2 cells (AT1/AT2) in the term distal lung just reach statistical overrepresentation. Other epithelial cells showing altered abundance in the term *CFTR*^*−/−*^ lung are secretory epithelial cells and basal cells, which are both substantially downregulated in the proximal lung alone. Importantly we validated the scRNA-seq data in the term proximal lung by quantitative immunostaining for ciliated cells, with an antibody specific for FOXJ1, and basal cells, with an antibody specific for KRT5. One potential concern relating to the immunochemistry data supporting the imbalance in FOXJ1+ ciliated and KRT5+ basal cells in the proximal term *CFTR*^*−/−*^ lung, is that these cell types are known to have different distributions along the length of the airways. Hence, our observations could also be accounted for if airways of different diameters/regions were examined in the WT and *CFTR*^*−/−*^ animals. However, we consider this extremely unlikely as the same pathologist harvested all the sheep lung tissues to ensure that equivalent anatomical regions of the lung were harvested from all sheep (see Fig. S1). Furthermore, at least 5 bronchioles within a consistent diameter range were measured on each slide from each of three WT and *CFTR*^*−/−*^ animals.

Next, focusing on immune cell populations, although the error bars in the single cell proportions test are large, T cells and B cells are significantly underrepresented in both regions of the *CFTR*^*−/−*^ lung at 80 days. In contrast at 120 days T cells and B cells are significantly overrepresented in proximal and distal *CFTR*^*−/−*^ lung respectively. At term T cells are underrepresented in both regions of the *CFTR*^*−/−*^ lung, while mast cells are also less abundant in the proximal lung as are monocytes and alveolar macrophages in the distal lung. However, since the immune cell clusters are among the smallest cell classes in the scRNA-seq data they may be readily biased, for example by an unusual distribution from individual animals. The underrepresentation of immune cells in both proximal and distal lung of *CFTR*^*−/−*^ animals at term warrants further investigation as it may suggest a delayed differentiation of the immune system in these animals. Alternatively, immune cell exhaustion might be occurring after earlier inflammation in the *CFTR*^*−/−*^ lung, though this is considered unlikely and lacks supporting data. Also of interest was whether in the scRNA-seq data we could identify the cellular signature of a transiently elevated inflammatory response that was seen between 100- and 120-days gestation only in *CFTR*^*−/−*^ distal lung in our previous bulk RNA-seq data (Kerschner et al. 2023). However, these two data sets are not directly comparable. Feature plots for DEGs that contributed to the inflammatory signature in the bulk data showed they were expressed in several cell types at 120 days, for example *KNG1* was most highly expressed in a subset of secretory epithelial cells, *CHI3L1* and *CXCL8* were seen primarily in a subset of myeloid cells and C3 was expressed in several cell types (data not shown). Further, none of the DEGs were seen on the marker gene lists in the scRNA-seq data. Further investigation of this transient early inflammatory response is needed to reveal underlying mechanisms.

One concern in interpretation of the results from proximal and distal lung at term relates to the rather stochastic nature of the birth of lambs born naturally rather by C-section. Our protocol relies on biological time of delivery for natural pregnancies though for cloned animals labor is induced on the calculated term date and lambs are usually delivered within 48 h. Since all naturally bred *CFTR*^*−/−*^ animals were genotyped at birth to confirm phenotypic predictions there was necessarily a variable delay between birth and euthanasia. The age of "term" animals ranged from 5.5 h to 72 h and this included newborns through to early neonates (Table 1). Earlier scRNA-seq studies in the CF pig collected tissues within a 24 h window after birth with no precise time of euthanasia available for each animal (Thurman et al. 2022). We attempted to age-match term WT and *CFTR*^*−/−*^ animals so that each group contained approximately the same distribution of hours post-delivery (Table 1). However, though sample distribution by postnatal age considering 3 groups (≤ 9 h; 10 −18 h and 24 −72 h) was similar in WT and *CFTR*^*−/−*^ distal lung, in proximal lung the distribution was less well balanced with relatively more *CFTR*^*−/−*^ samples before 18 h). Of note, the sheep fetal lung undergoes major alterations at birth, when it changes from a fluid-filled to an air-filled organ. However, the physiological processes that accompany fluid removal, including a shift from epithelial cell chloride secretion to sodium absorption, commence during labor and are complete within ~ 2 h of birth (Morton and Brodsky 2016). Moreover, these processes are not known to be associated with alterations in the frequency of differentiated cell types as we observe here between WT and *CFTR*^*−/−*^ lung tissues, which are likely to develop over a much longer time window.

Another topic that warrants discussion is whether the cloning (SCNT) process used to generate *CFTR*^*−/−*^ animals contributes to the cellular imbalances we observe. Epigenetic changes in cloned animals were reported previously although not specifically affecting lung tissues (Wang et al. 2020). With respect to the cellular imbalance that we observe in *CFTR*^*−/−*^ proximal and distal lung tissues at term we can be certain that this is not an artifact of SCNT, since all but one lamb investigated at term were the outcomes of natural breeding of *CFTR*^+/−^ ewes and rams. Furthermore, the UMAP plot of cell clusters by donor (Fig. S2) does not show any unusual features in cells from the cloned animal and their removal from the single cell proportions test did not alter the output. All 80 days *CFTR*^*−/−*^ animals in this cohort are cloned, so we cannot currently exclude epigenetic artifacts. However, since we draw few conclusions from this timepoint due to the challenges of cell identification this is not considered a major deficit. At 120 days though the majority of *CFTR*^*−/−*^ animals are cloned we also include 2 *CFTR*^*−/−*^ lambs arising from natural breeding, which were detected by in utero genotyping that was confirmed at collection. Inclusion or exclusion of scRNA-seq data from these 2 animals had little impact on the results. Since future generations of *CFTR*^*−/−*^ sheep will all be bred naturally, we expect to resolve any doubts on this topic.

Lastly, we compared our results to the scRNA-seq analysis of human end-stage CF lung tissue and controls (Carraro et al. 2021), which to date is the most comprehensive study of lung disease at single cell resolution in pwCF. This analysis also reported altered cellularity in the CF tissue, including an overabundance of epithelial cells transitioning to specialized ciliated and secretory cell subsets together with a decrease in cycling basal cells. Our observation of overrepresentation of ciliated cells in the sheep *CFTR*^*−/−*^ proximal and distal lung at term and underrepresentation of basal cells in the proximal lung are consistent and may be indicative of earlier stage disease contributing to this phenotype. This raises the question how this overrepresentation of ciliated cells might influence the course of early lung disease in babies with CF.

In much earlier work (Coates et al. 1984) examined the age-related susceptibility of WT ferrets to influenza virus and found that newborn ferrets died following intranasal inoculation of influenza virus while 15-day old pups were nearly as resistant as adult ferrets to the same protocol. The newborn animals primarily succumbed to lower respiratory tract disease. Close examination of the cellularity of the lower respiratory tract lung epithelium at each time point showed a substantial difference in the ratio of ciliated epithelium to alveolar epithelium, with a two-fold higher ratio in the newborns compared to 15-day old pups and a four-fold higher ratio compared to adults. Though there are also many immunological differences between newborn and older ferrets this alteration in epithelial cell ratios was proposed as a contributing mechanism. We specifically compared the ratios of ciliated cells to alveolar cell types in *CFTR*^*−/−*^ and WT lambs at term and noted a trend of consistent increase in this ratio in our scRNA-seq data, though the increase reached statistical significance only comparing ciliated cells and AT2 cells in the distal lung (Fig. S10). These observations would support a hypothesis that the increase in ciliated cells at term in *CFTR*^*−/−*^ sheep lung could contribute to early respiratory pathogen susceptibility in newborn lambs. This hypothesis warrants further investigation both in the sheep CF model and in human, where early viral infections may contribute to airway inflammation in infants with CF (Deschamp et al. 2019).

## Supplementary Information

Supplementary Material 1.

Supplementary Material 2.

Supplementary Material 3.

Supplementary Material 4.

Supplementary Material 5.

Supplementary Material 6.

Supplementary Material 7.

Supplementary Material 8.

Supplementary Material 9.

Supplementary Material 10.

## Data Availability

All sequencing data from this project are deposited at GEO: GSE281174.
