# Supplementary material for: Cellular imbalance in proximal and distal lung of CFTR−/− sheep in utero and at birth
Source: Mol Med. 2025 Jun 11;31:231. doi: 10.1186/s10020-025-01266-7 (PMC12153128; doi:10.1186/s10020-025-01266-7)
Supplement: Supplementary file 1 — Supplementary Material 1. [file 10020_2025_1266_MOESM1_ESM.pdf]

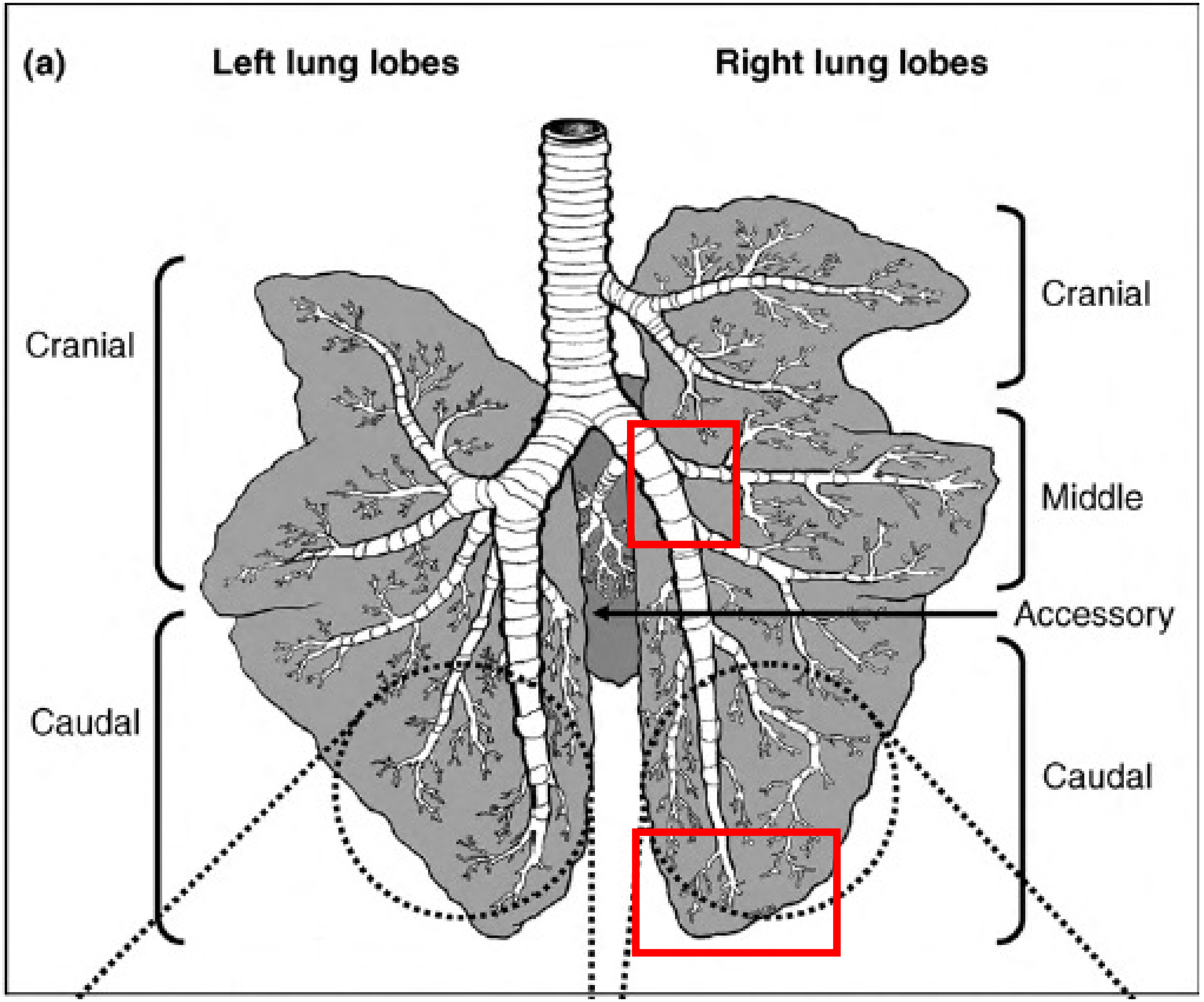

• Image from 10.1016/j.ddmod.2009.12.002

Figure S1

Proximal and distal lung tissue collection sites shown as red boxes.

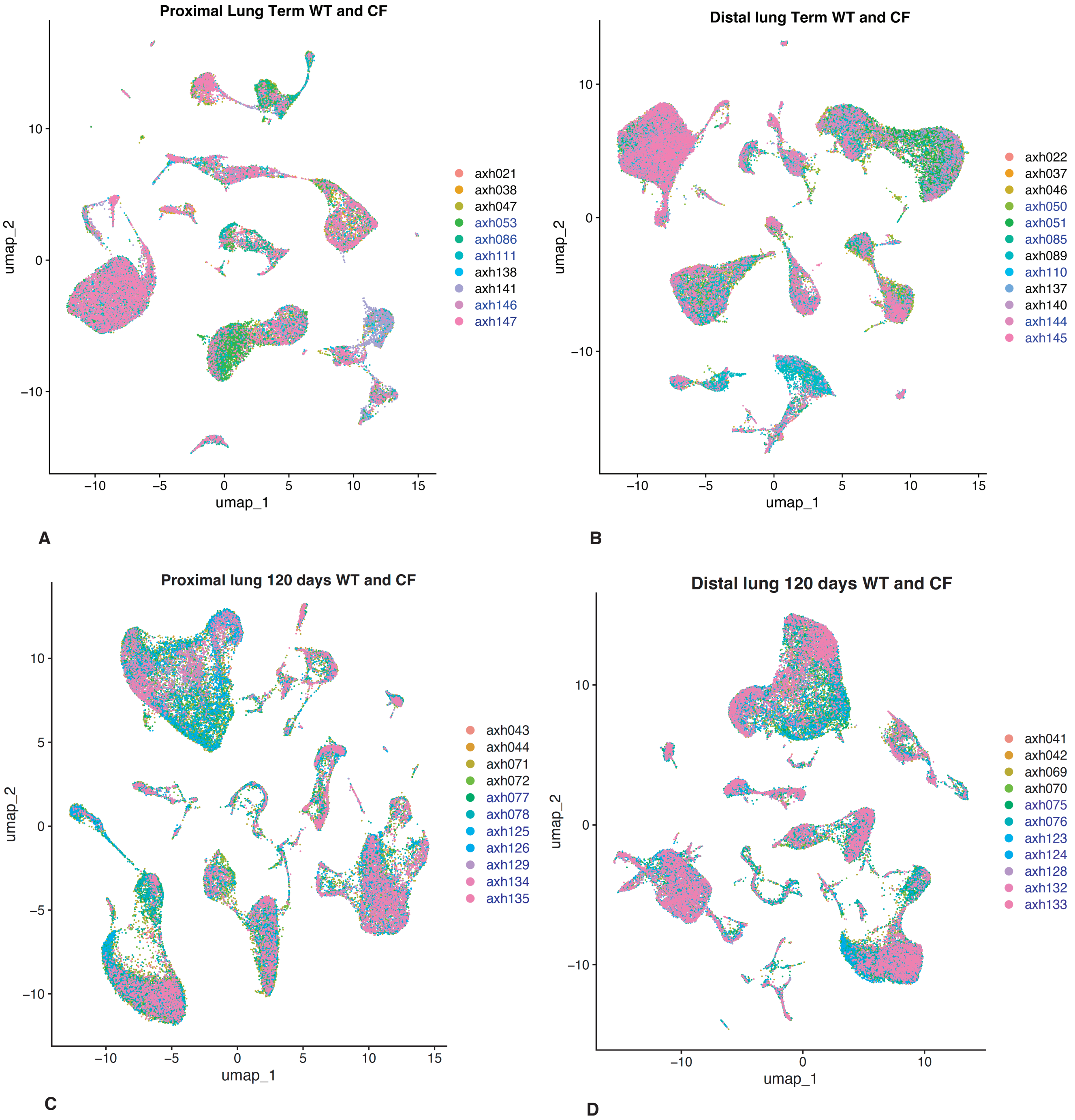

Figure S2

UMAP by donor for Proximal and Distal lung at Term (A,B) and 120 days (C,D). Samples in black font are WT and in blue font CF. All *CFTR*$^{-/-}$ tissues at term are from naturally bred lambs except axh144-147, which are from cloned *CFTR*$^{-/-}$ lambs. All *CFTR*$^{-/-}$ tissues at 120 days are from cloned animals except axh132-135, which are naturally bred.

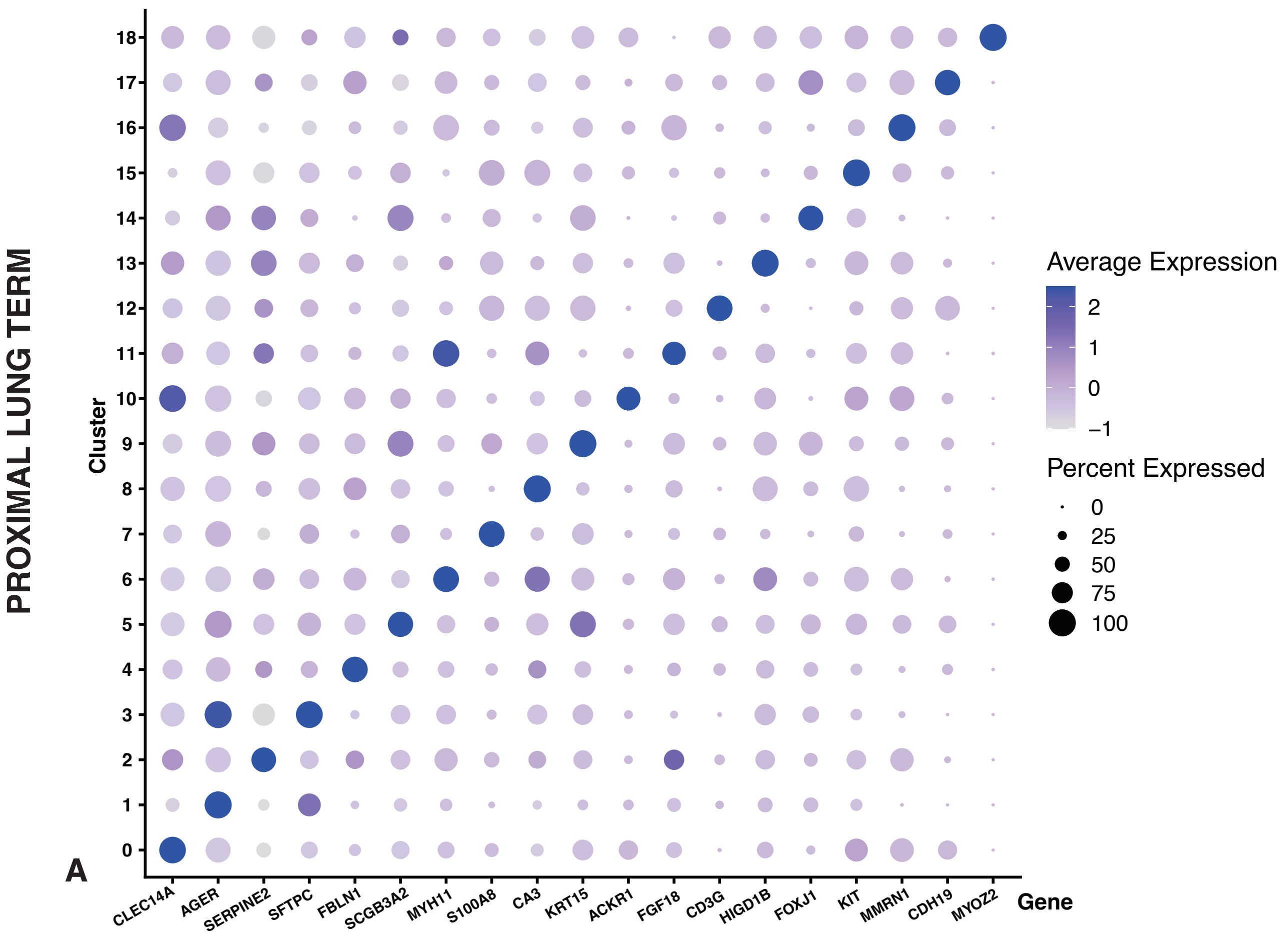

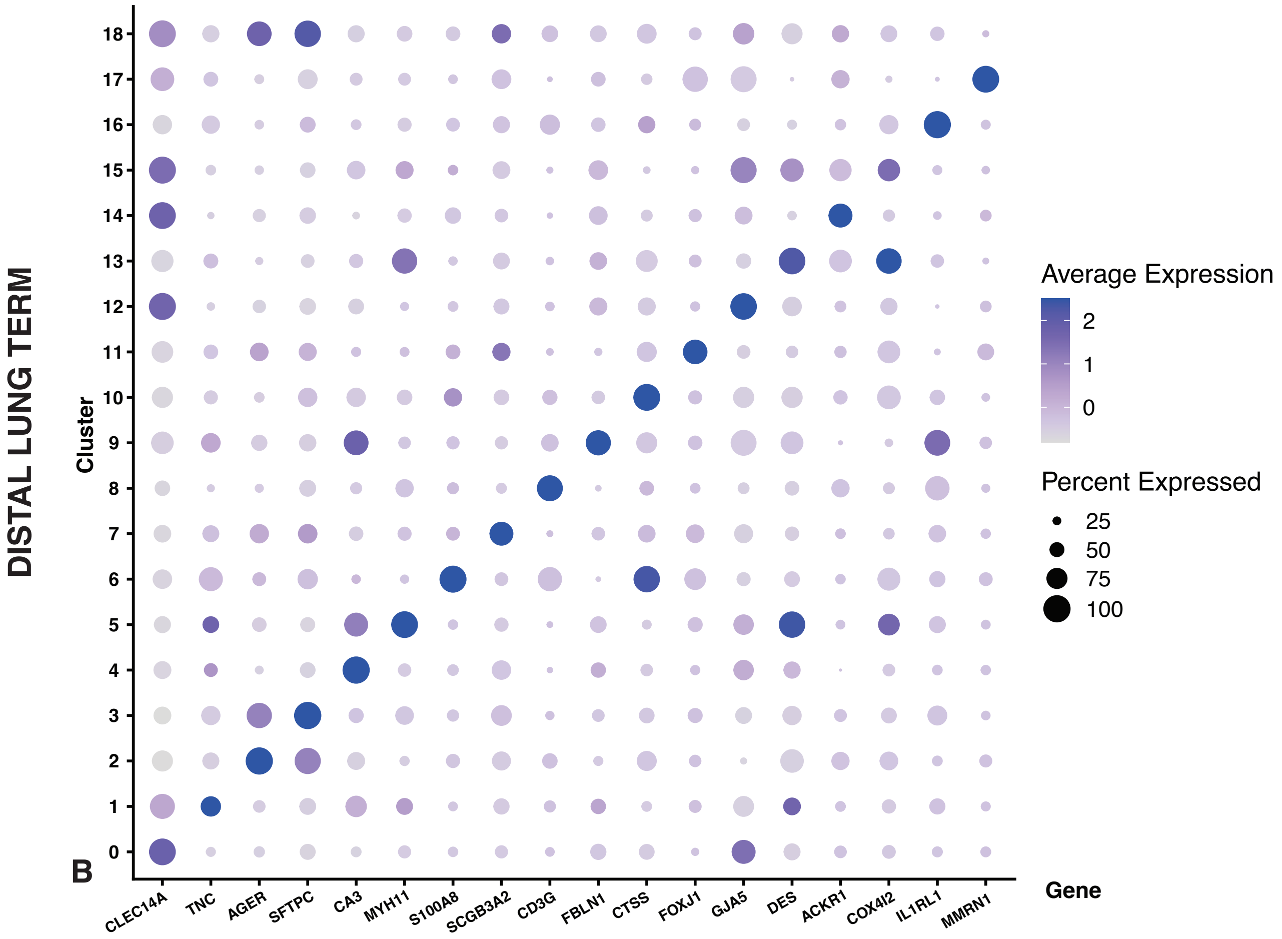

Figure S3

Dot plots show marker gene expression by cluster in A) Proximal Lung and B) Distal Lung at term.

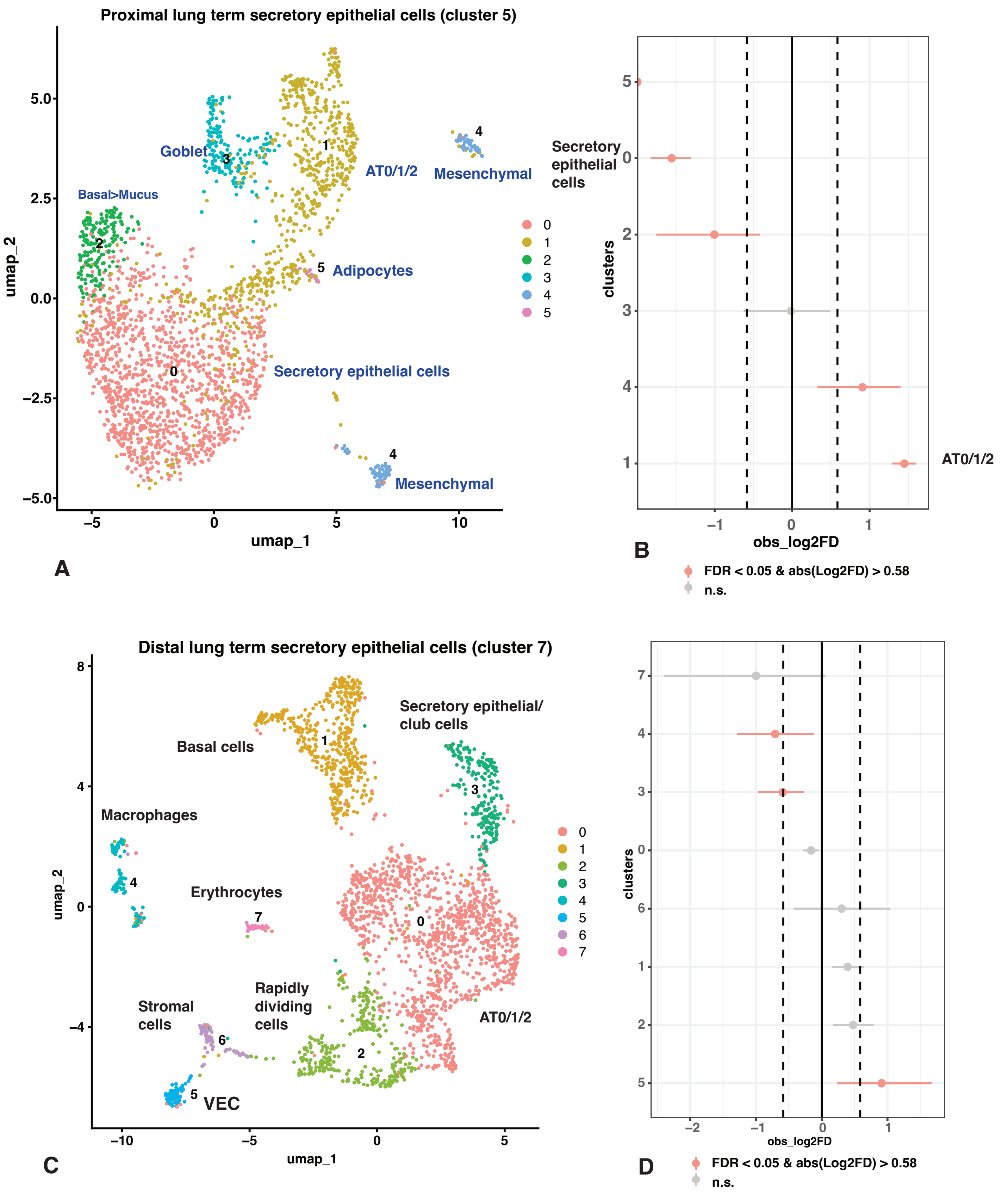

Figure S4 Subclustering of epithelial cells (Fig. 1 Cluster 5 and Fig. 2 Cluster 7) from Proximal and Distal Term UMAPs respectively shown in Fig.1 and Fig. 2. A. Cluster 5 UMAP plot shows 6 cell types with identities assigned from the marker gene list in Table S2A. **B.** The single cell proportions test shows that within this epithelial cell compartment, AT0/1/2 cells are overrepresented in *CFTR*$^{-/-}$ proximal term lung and secretory epithelial cells are underrepresented. **C.** Cluster 7 UMAP plot shows 8 cell types with identities assigned from the marker gene list in Table S2B. D. The proportions test suggests no real differences between *CFTR*$^{-/-}$ and WT though 3 clusters just reach statistical significance.

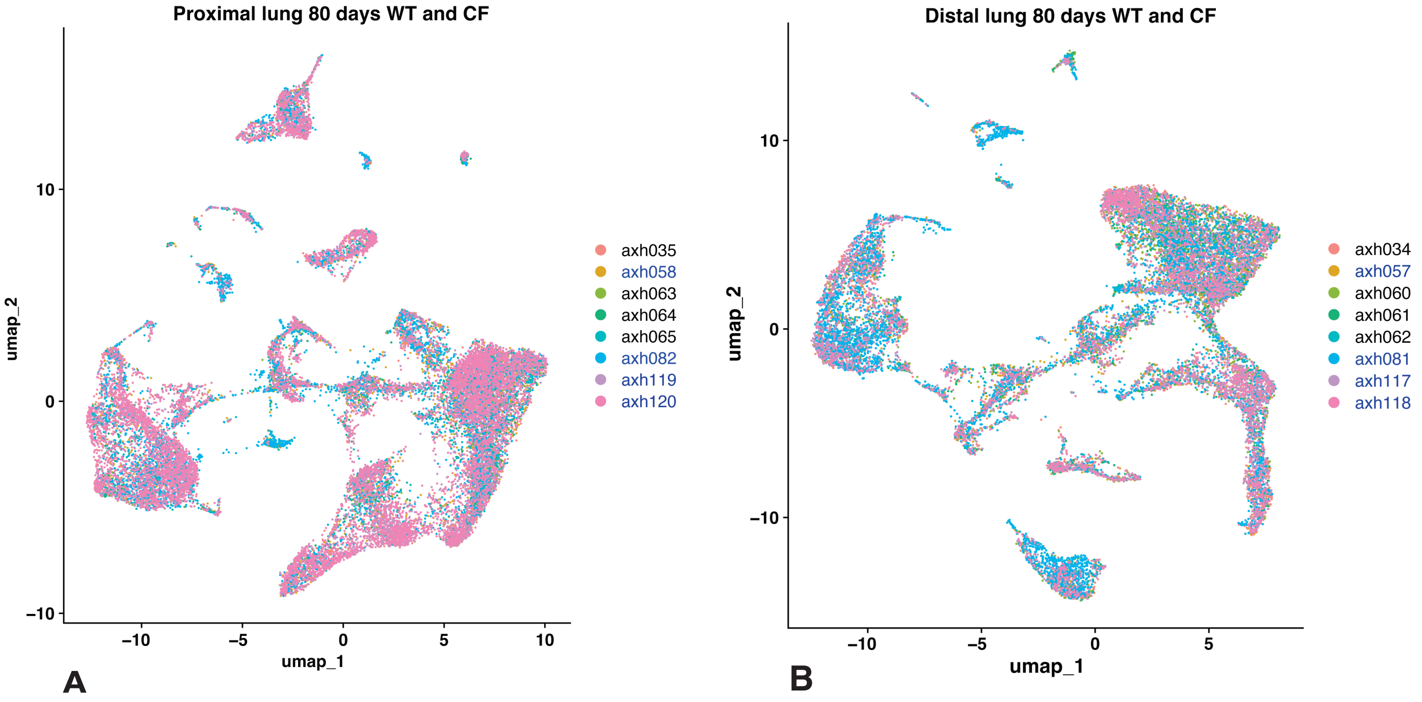

Figure S5

UMAP by donor for Proximal (A) and Distal (B) lung at 80 days. Samples in black font are WT and in blue font CF. All *CFTR*$^{-/-}$ tissues are from cloned lambs.

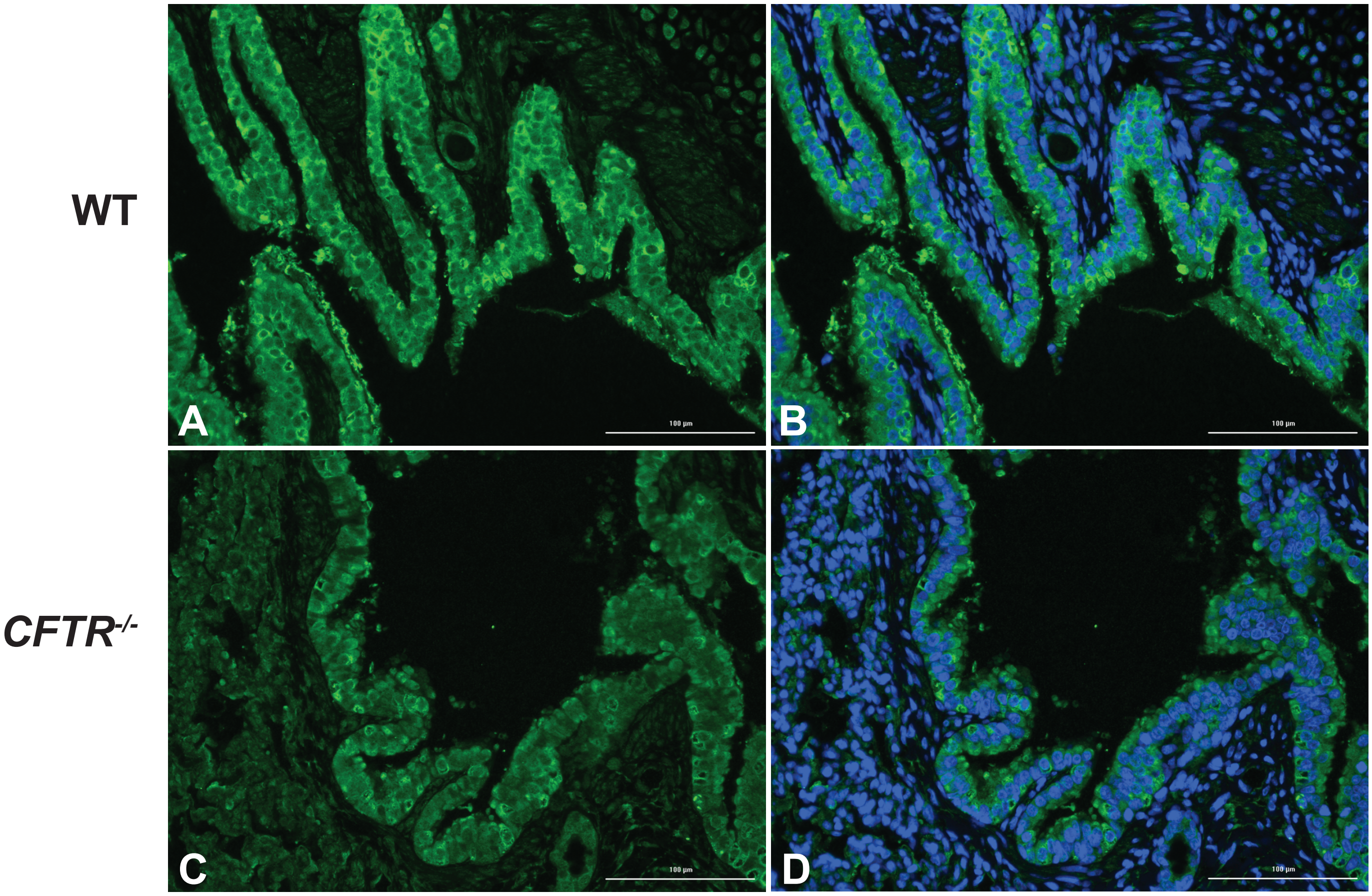

Figure S6. Underrepresentation of SCGB3A2-expressing secretory epithelial cells in bronchioles of *CFTR*$^{-/-}$ proximal lungs.

Representative images show SCGB3A2 expression in the bronchioles of proximal lung sections from WT (A, B) and *CFTR*$^{-/-}$ (C, D) animals. (A, C) SCGB3A2 staining is shown in green; (B, D) overlay images show SCGB3A2 staining and the DAPI nuclear counterstain in blue. Scale bar = 100 µm.

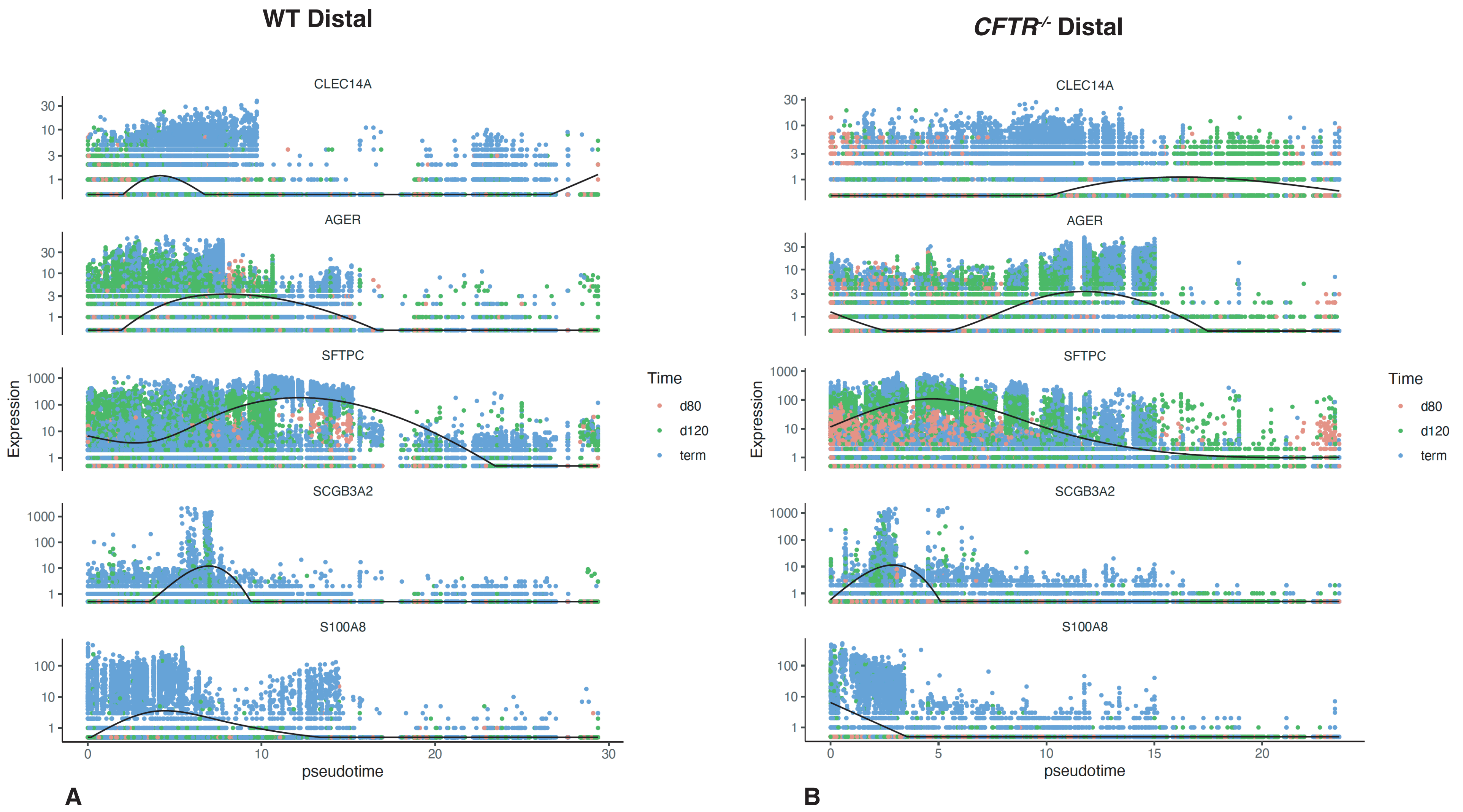

Figure S7

Pseudotime analysis of scRNA-seq data using Monocle 3 shows altered gene expression profiles in WT and *CFTR* $^{-/-}$ sheep distal lung. Markers for AT1 cells (AGER), AT2 cells (SFTPC), secretory epithelial cells (SCGB3A2) and myeloid cells (S100A8) show rather similar splines in WT and CFTR -/- tissues, while a marker for VECs (CLEC14A) has differejt profiles in the two genotypes.

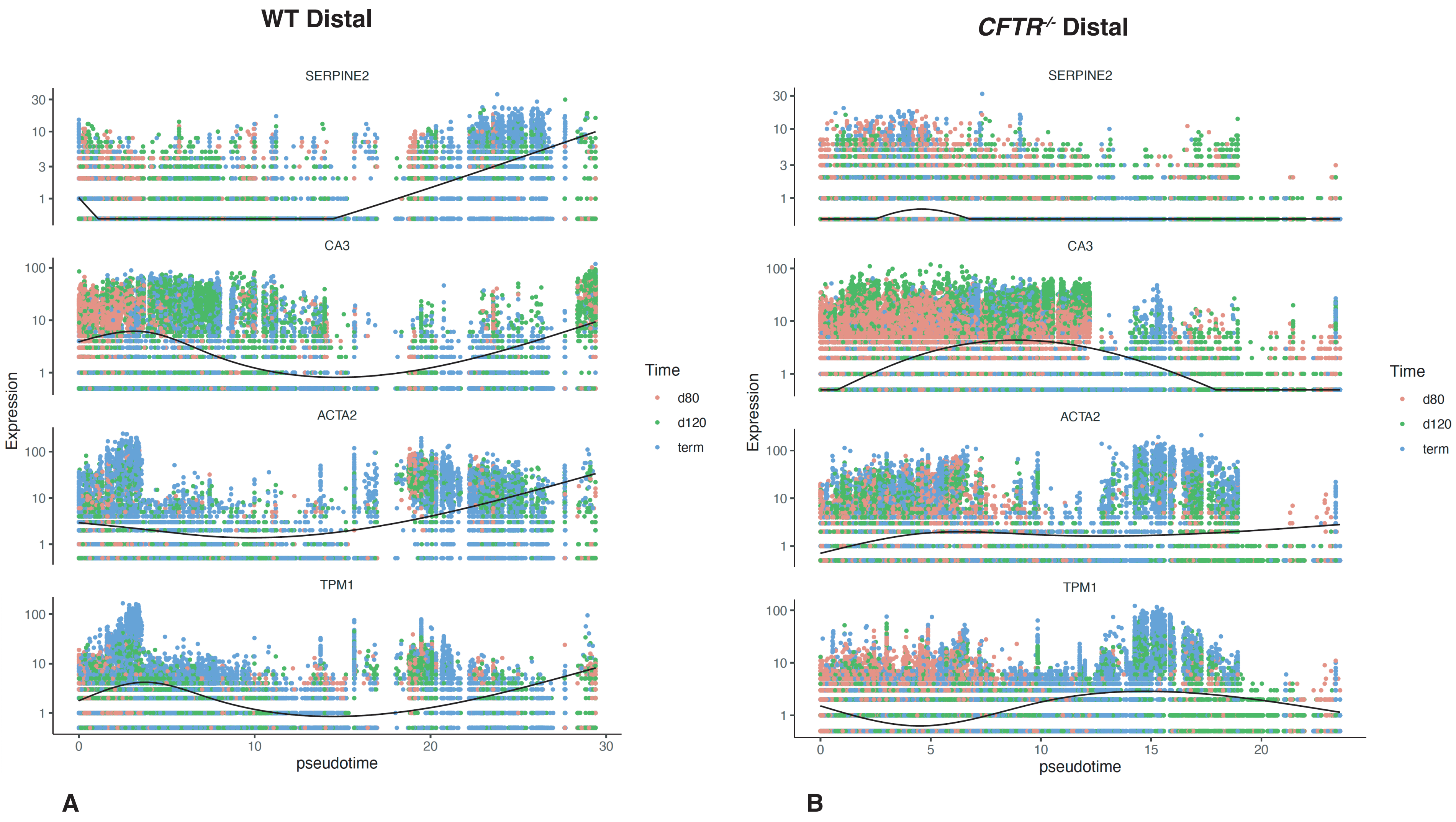

Figure S8

Pseudotime analysis of scRNA-seq data using Monocle 3 shows altered gene expression profiles in WT and *CFTR* $^{-/-}$ sheep distal lung. Markers for SCMF (*SERPINE2*), AF1 cells (*CA3*), ASMC (*ACTA2*) and VSMC (*TPM1*) show splines with very different, almost reciprocal profiles in the WT and *CFTR* $^{-/-}$ animals.

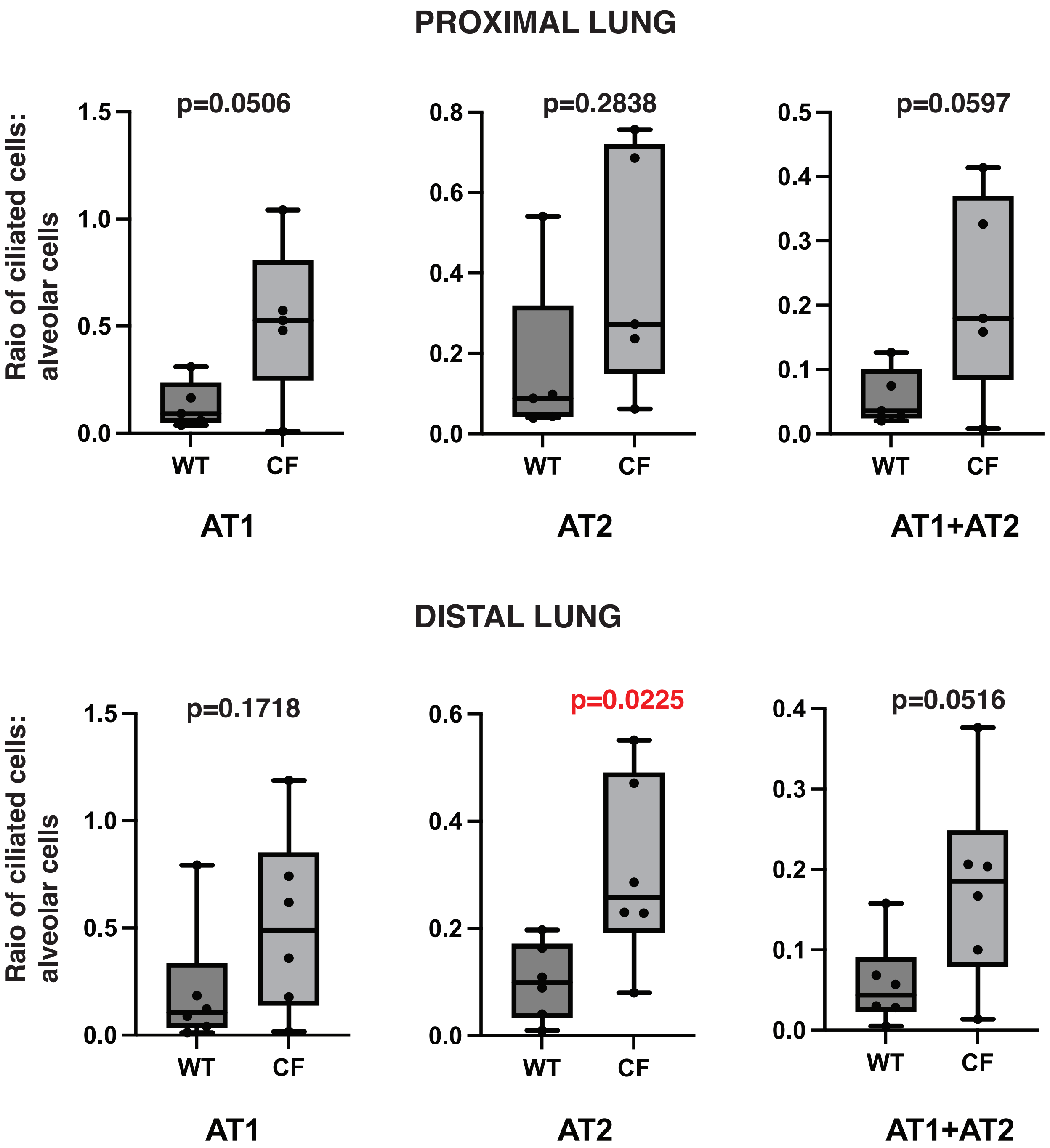

Figure S9

Ratios of ciliated cells to alveolar cells in proximal and distal lung at term in WT and *CFTR*$^{-/-}$ (CF) sheep.

Proximal cluster 14 to 1, 3, or 1 + 3. Distal cluster 11 to 2, 3, or 2 + 3.
Statistics:unpaired t-test.
