# Supplementary material for: Cellular imbalance in proximal and distal lung of CFTR−/− sheep in utero and at birth
Source: Mol Med. 2025 Jun 11;31:231. doi: 10.1186/s10020-025-01266-7 (PMC12153128; doi:10.1186/s10020-025-01266-7)
Supplement: Supplementary file 2 — Supplementary Material 2. [file 10020_2025_1266_MOESM2_ESM.pdf]

Methods

Sex as a biological variable.

Our study examined male and female animals where possible since all cloned animals used here are male. Results are combined and sex was not considered as a biological variable.

The materials and methods for this study were as described in our previous work (Van Wettere et al. 2022; Kerschner et al. 2023; Leir et al. 2024), but are reiterated here for purposes of reproducibility.

Animals. American Romney breed of domestic sheep (*Ovis aries*) was used in this study. All animal studies were approved and monitored by the Institutional Animal Care and Use Committee (IACUC) at Utah State University (IACUC protocol # 10089) and conformed to the National Institute of Health guidelines. WT sheep were bred according to standard protocols. Briefly, ewes were synchronized at estrus using an intramuscular (IM) injection of 2.5 mL EstruMate containing 250 μg/ml of cloprostenol, a synthetic analogue of prostaglandin F2α (Merck Animal Health) or Controlled Internal Drug Release (CIDR) (0.3g prostaglandin) and introduced to the ram on the same day. Ultrasonography around 40 days confirmed pregnancy status and day 1 of gestation was defined as 48 hours post EstruMate injection. WT term animals were born naturally and time after birth until euthanasia was recorded. Additionally, heterozygous exon 2 targeted (Fan et al. 2018) ($CFTR^{+/-}$) ewes were bred to an exon 2 targeted $CFTR^{+/-}$ ram to produce $CFTR^{-/-}$ lambs by natural breeding at 120 days or at natural term. All lambs were genotyped immediately

after birth using PCR/RFLP assay and Sanger sequencing as previously described (Fan et al. 2018) to confirm the *CFTR*$^{-/-}$ animals.

Generation of CFTR$^{-/-}$ *sheep pregnancies by somatic cell nuclear transfer (SCNT).*

For scRNA-seq experiments: a male neonatal fibroblast cell line (CF2503, exon 2 targeted *CFTR*$^{-/-}$) was used for SCNT as described previously (Fan et al. 2018). In total, 290 cloned embryos were transferred into 22 estrus synchronized recipients. Eleven pregnancies were initially established as confirmed by ultrasonography at 40±3 days of gestation (11/22=50%). The samples collected at 80 days, 120 days gestation or at term are summarized in Table 1. As previously PCR-RFLP and Sanger sequence results (Fan et al. 2018) indicated that all cloned fetuses/lambs carried the same mutations as those of the donor cells they originated from.

Histopathologic analysis and immunochemistry. A necropsy was performed on all fetuses collected, to examine for gross lesions and the findings were documented as described previously (Van Wettere et al. 2022). Lung tissue samples were collected and fixed in 10% neutral buffered formalin for histology. Formalin-fixed tissue sections were processed and embedded in paraffin according to routine histologic techniques. Sections, 5-μm thick, were stained with hematoxylin and eosin (H&E), alcian blue, or periodic acid–Schiff (PAS) stain according to standard methods and examined by light microscopy. For immunostaining of sections, tissues were deparaffinized and rehydrated, followed by antigen retrieval with sodium citrate buffer (10 mM sodium citrate and 0.05 % Tween 20, pH 6.0) in a 98 °C water bath for 45 min. The sections were then post-fixed in 4 % paraformaldehyde (in PBS) for 15 min, permeabilized with 0.5 % saponin for 10 min, blocked with 3 % BSA, and stained by standard protocols. Briefly, the sections were first incubated with primary

antibodies FOXJ1 (Abcam ab235445), KRT5 (Invitrogen MA5-16372) and SCGB3A2 (abcam ab181853) all at 1/100 dilution in PBS containing 0.1% BSA] overnight at 4°C. After three washes with PBS containing 0.05% tween 20 (PBS-T), the sections were incubated with the secondary antibody Alexa Fluor 488 AffiniPure Goat Anti-Rabbit IgG (H+L) (Jackson ImmunoResearch) at 1/800 dilution, at room temperature for 1 hr in the dark. After three further washes with PBS-T, the specimens were counterstained with DAPI and mounted with mounting medium (Vector laboratories H-1700). Images were captured by the Lionheart FX Automated Microscope (BioTek) and cell quantification performed using Gen5 software (BioTek). Briefly, at least five bronchioles were randomly selected from each sample, the epithelium was delineated manually and then cells within the delineation were counted automatically. The percentages of FOXJ1-positive apical ciliated epithelial cells and KRT5-positive basal cells were calculated by dividing the number of FOXJ1- or KRT5-positive cells by the total number of DAPI-positive apical or basal cells, respectively. Proximal lung sections from three different term WT and CF animals were analyzed. The results represent a total of >1100 epithelial cells analyzed for FOXJ1 and > 800 epithelial cells analyzed for KRT5.

Single-cell RNA sequencing (scRNA-seq).

1) Isolation of single cells from tissues. Tissues for scRNA-seq were from WT animals at 80-, 120- and 147 days (term) gestation, cloned *CFTR*$^{-/-}$ animals at 80-, 120-days and term, and naturally bred *CFTR*$^{-/-}$ lambs at 120 -days and term. For single cell isolation from proximal or distal lung (regions shown in Fig. S1) ~ 300 mg of fresh tissue was cut into 2-3 mm pieces and subjected to two rounds of digestion. First in 5ml of collagenase solution (200 U/ml collagenase, 40 IU/ml DNase I (all from Worthington), 3 mM $CaCl_2$, at 37°C, with

continuous stirring for 30-40 min. Alternatively, Roche Liberase TL (Millipore Sigma) containing 100 µg/ml collagenase was used. Tissue clumps were then disassociated by pipetting 10 times in a 5 ml serological pipette and left to settle for 3-4 min at which point the supernatant was collected and stored on ice as cell suspension 1. Second, 5 ml of fresh collagenase digestion solution as above was added to the settled tissue clumps remaining after the first digestion and again stirred at 37°C for 30-40 min, at which point very little tissue debris remained. The supernatant (cell suspension 2) was pooled with cell suspension 1 and together passed through a cell strainer (200 µm, pluriSelect) to remove any tissue debris. The strained cell suspension was then centrifuged at 300 x g for 5 min, washed once in HBSS (Sigma Aldrich) + 2% FBS and centrifuged again. 4 ml of 1× Red Blood Cell Lysis Solution (Miltenyi Biotec 130-094-183) was added to the cell pellet which was vortexed for 5 seconds and then incubated for 5 minutes at room temperature. After 10 ml of HBSS with 2% FBS was added, the mixture was centrifuged at 300 × g for 5 min and the supernatant was removed. The cell pellet was then digested with 3 ml of Accutase (Stem Cell Technologies, #AT104) for 15-25 min with frequent gentle pipetting with a wide-bore pipette after which time cells were visualized under a microscope. If any cell clumps persisted these were collected by centrifugation and further digested with 3 ml of Accutase for an additional 10-15 min. Accutase was removed by washing the cells with 5 ml HBSS + 2% FBS, followed by centrifugation to collect the cells. The supernatant was discarded, and the cell pellet was resuspended in 0.5-1.0 ml HBSS, passed through a 20-µm mini cell strainer (pluriSelect) and cells counted.

2) Single-cell RNA-sequencing and analysis

i) Approximately 3000- 5000 cells were used for scRNA-seq using the 10x Genomics Chromium Single Cell 3' Reagent Kit v3, or v3.1. After quality control of both cDNA and final libraries using Tapestation, the libraries were sequenced on a NovaSeq 6000 machine. Reads were aligned to the Oar_v4.0/oviAri4 (Texel) genome using Cell Ranger 3.1.0. Cells were filtered for quality using cuts for standard metrics: library size, number of detected genes and mitochondrial read percentage (see Table S1 for cut values for each sample). Ribosomal protein genes were also filtered out. The resulting objects were normalized and batch-corrected using the Seurat R package (Hao et al. 2021), version 5.1.0, followed by clustering and UMAP (Uniform Manifold Approximation and Projection) dimensionality reduction. Seurat was also used to find cluster markers by performing differential gene expression analysis between clusters using the receiver operating characteristic (ROC) method. To compare cell proportions between *CFTR*$^{-/-}$ and WT in each cluster, a monte-carlo/permutation test was performed using the scProportionTest R package (https://github.com/rpolicastro/scProportionTest) (Miller et al. 2021).

ii) *Pseudotime analysis* was performed in R (version 4.4.1) using the monocle3 package (version 1.4.17) (https://cole-trapnell-lab.github.io/monocle3). Twenty seven wild type (WT) (4 distal and 4 proximal at 80-days, 4 distal and 4 proximal at 120-days of gestation and 6 distal and 5 proximal at term) and thirty three *CFTR*$^{-/-}$ (4 distal and 4 proximal at 80-days, 7 distal and 7 proximal at 120-days of gestation and 6 distal and 5 proximal at term) samples were separately run through the same pipeline.

For each of the samples, contents of the *filtered_gene_bc_matrices* directory of the *cellranger count* command were used as an input and converted to a Monocle3 object using the *load_mm_data* command. Then samples for each of the runs (WT or *CFTR*$^{-/-}$) were combined for each time point and each lung location, the resulting object normalized by log and size factor, and the lower dimensional space calculated using the PCA method. The number of principal components (PC) to use in downstream analysis was found using an elbow plot, resulting in 30 PC used for *CFTR*$^{-/-}$ proximal samples, 28 PC used for WT proximal samples, 27 PC used for *CFTR*$^{-/-}$ distal samples, and 24 PC used for WT distal samples.

The samples were consequently "aligned" to compensate for batch effects using the *align_cds* command and UMAP reduced dimensions calculated. Clustering was then performed using the *cluster_cells* command with a resolution of 1e-6 and 20 nearest neighbors for *CFTR*$^{-/-}$ proximal and distal and WT proximal samples, and a resolution of 1e-5 and 20 nearest neighbors for WT distal samples and the "trajectory" of gene expression change (pseudotime trajectory) found using the *learn_graph* command with default arguments, thus learning a disjointed graph in each partition.

To find pseudotime start points, starting principal points (root principal nodes) were manually identified by picking root nodes in each partition with the largest percentage of earlier gestation time point cells surrounding them. Then, cells were ordered in pseudotime using the found principal nodes using the *order_cells* command.

Fan Z, Perisse IV, Cotton CU, Regouski M, Meng Q, Domb C, Van Wettere AJ, Wang Z, Harris A, White KL et al. 2018. A sheep model of cystic fibrosis generated by CRISPR/Cas9 disruption of the CFTR gene. *JCI Insight* **3**: e123529.

Hao Y, Hao S, Andersen-Nissen E, Mauck WM, 3rd, Zheng S, Butler A, Lee MJ, Wilk AJ, Darby C, Zager M et al. 2021. Integrated analysis of multimodal single-cell data. *Cell* **184**: 3573-3587 e3529.

Kerschner JL, Paranjapye A, Schacht M, Meckler F, Huang F, Bebek G, Van Wettere AJ, Regouski M, Perisse IV, White KL et al. 2023. Transcriptomic analysis of lung development in wildtype and CFTR(-/-) sheep suggests an early inflammatory signature in the CF distal lung. *Funct Integr Genomics* **23**: 135.

Leir SH, Tkachenko S, Paranjapye A, Meckler F, Van Wettere AJ, Kerschner JL, Kuznetsov E, Schacht M, Gillurkar P, Regouski M et al. 2024. Stellate cells are in utero markers of pancreatic disease in cystic fibrosis. *Mol Med* **30**: 115.

Miller SA, Policastro RA, Sriramkumar S, Lai T, Huntington TD, Ladaika CA, Kim D, Hao C, Zentner GE, O'Hagan HM. 2021. LSD1 and Aberrant DNA Methylation Mediate Persistence of Enteroendocrine Progenitors That Support BRAF-Mutant Colorectal Cancer. *Cancer Res* **81**: 3791-3805.

Van Wettere AJ, Leir SH, Cotton CU, Regouski M, Perisse IV, Kerschner JL, Paranjapye A, Fan ZQ, Liu Y, Schacht M et al. 2022. Early developmental phenotypes in the cystic fibrosis sheep model. *Faseb Bioadvances* **5**: 13–26.
